# The Th17/IL-17 Axis and Kidney Diseases, With Focus on Lupus Nephritis

**DOI:** 10.3389/fmed.2021.654912

**Published:** 2021-09-03

**Authors:** Feliciano Chanana Paquissi, Hugo Abensur

**Affiliations:** ^1^Department of Medicine, Clínica Girassol, Luanda, Angola; ^2^Faculdade de Medicina, Universidade de São Paulo, São Paulo, Brazil; ^3^Hospital das Clínicas, Faculdade de Medicina, Universidade de São Paulo, São Paulo, Brazil

**Keywords:** lupus nephritis, Th17 cells, inflammation, fibrosis, interleukin-17

## Abstract

Systemic lupus erythematosus (SLE) is a disease characterized by dysregulation and hyperreactivity of the immune response at various levels, including hyperactivation of effector cell subtypes, autoantibodies production, immune complex formation, and deposition in tissues. The consequences of hyperreactivity to the self are systemic and local inflammation and tissue damage in multiple organs. Lupus nephritis (LN) is one of the most worrying manifestations of SLE, and most patients have this involvement at some point in the course of the disease. Among the effector cells involved, the Th17, a subtype of T helper cells (CD4+), has shown significant hyperactivation and participates in kidney damage and many other organs. Th17 cells have IL-17A and IL-17F as main cytokines with receptors expressed in most renal cells, being involved in the activation of many proinflammatory and profibrotic pathways. The Th17/IL-17 axis promotes and maintains repetitive tissue damage and maladaptive repair; leading to fibrosis, loss of organ architecture and function. In the podocytes, the Th17/IL-17 axis effects include changes of the cytoskeleton with increased motility, decreased expression of health proteins, increased oxidative stress, and activation of the inflammasome and caspases resulting in podocytes apoptosis. In renal tubular epithelial cells, the Th17/IL-17 axis promotes the activation of profibrotic pathways such as increased TGF-β expression and epithelial-mesenchymal transition (EMT) with consequent increase of extracellular matrix proteins. In addition, the IL-17 promotes a proinflammatory environment by stimulating the synthesis of inflammatory cytokines by intrinsic renal cells and immune cells, and the synthesis of growth factors and chemokines, which together result in granulopoiesis/myelopoiesis, and further recruitment of immune cells to the kidney. The purpose of this work is to present the prognostic and immunopathologic role of the Th17/IL-17 axis in Kidney diseases, with a special focus on LN, including its exploration as a potential immunotherapeutic target in this complication.

## Introduction

Systemic lupus erythematosus (SLE) is a disease characterized by hyperreactivity to the self, with the polarization of the immune response to a proinflammatory profile (1, 2), autoantibodies production (3), immune complex formation (4) and deposition in tissues (5). It also occurs with local production of inflammatory mediators, and additional recruitment of inflammatory cells, resulting in tissue damage in various organs (6). These events together express the dysregulation of the local and systemic immune response, which characterize the disease (1, 7, 8). Lupus nephritis is one of the most worrying organic affectations of lupus, one of the strongest predictors of a poor outcome in SLE, being responsible for the greater burden attributable to the disease, mainly in the low-income populations (9, 10).

Several immunological pathways are involved in the pathogenesis of SLE (11), which calls us to go deeper in the knowledge about the immunopathologic complexity of this disease, aiming to explore new opportunities for targeted therapies (12). Among the effector cells, the Th17, a subtype of T helper cells (CD4+), is one of those that has shown significant hyperactivation (1, 13–15); correlates with disease activity (1, 16, 17), being involved in many manifestations of SLE as neuropsychiatric (18, 19), cutaneous (20, 21), and lupus nephritis (1, 22, 23); and correlates with the fatality of the disease (24).

Th17 cells have IL-17A and IL-17F as major cytokines, and the Th17/IL-17 axis has dominantly an effector and proinflammatory functional profile (25, 26), being involved in the pathogenesis of many immune-mediated diseases (27–30). The receptors for the IL-17 family (IL-17RA, IL-17RC, IL-17RE) are expressed in most intrinsic kidney cells (podocytes, tubular epithelial, mesangial, and renal endothelial cells) (31–35), and are involved in the promotion of a proinflammatory environment, disruption in the morphology and function of nephron elements (36, 37), in the activation of many profibrotic pathways (36, 38), which results in fibrosis, loss of architecture (37, 39), and consequent loss of organ function (23, 40).

Other studies highlight the predominant role of the Th17/IL-17 axis in LN that even in models deficient in TNF receptors (another potent proinflammatory cytokine), Th17-associated pathways were sufficient to cause the clinical and pathological changes of lupus nephritis (41). In addition, other Th17-related elements of the immune response participate in this process (16, 42, 43). Thus, IL-23 (involved in the differentiation and maintenance of Th17 by an autocrine mechanism) is also increased in lupus and correlates with disease activity (44, 45). In experimental models, the RORγt (the Th17-defining transcription factor) promoted itself glomerulonephritis; and RORγt ablation or deficiency (RORγt–/–) conferred protection to experimentally induced glomerulonephritis (46, 47). This review focuses on the role of the Th17/IL-17 axis in the immunopathology and prognosis of lupus nephritis and its exploration as a potential immunotherapeutic target in this complication.

## Association Between Th17/IL-17 Axis and Lupus Nephritis Prognostic Factors

### Proteinuria, Hematuria, and Anemia

Serum IL-17 levels are significantly associated with proteinuria (48, 49); and its concentration at the baseline keeps a positive correlation with the severity of proteinuria (50). In a study involving 15 patients (who underwent kidney biopsy), using the laser microdissection technique, the percentage of IL-17+ TCR+ among kidney-infiltrating cells correlated positively with hematuria in LN (51). In another study, elevated serum levels of IL-17 and IL-6 were associated with anemia (52).

### Severity Scores and Histological Activity

Th17 cell frequencies significantly correlated with SLEDAI and inversely with C3 (53) and the concentration of IL-17 at the baseline kept a positive correlation with other parameters of severity (ESR, SLEDAI scores, and ANA titers) (50). In relation to histological activity, Th17 cell frequencies in peripheral blood and IL-17 levels in serum correlated significantly with renal biopsy classification for LN (43, 49, 53). A significant positive association has been found between serum IL-17 (and TWEAK) levels and nephritis activity index (54). In another study, the frequencies of circulating Th17-cells correlated positively with histological activity index, cellular crescent, and endocapillary proliferation. Additionally, intraglomerular levels of IL-17 and IL-23 were significantly higher in class IV LN than in MCN patients or HC (43). In another study, with measurement of urinary IL-17 (uIL-17), the levels of uIL-17 were significantly higher in the severe LN than in the control group (*P* < 0.05); and increased with disease severity seen in biopsy (mean ± SD: 43.96 ± 24.04, 55.69 ± 33.21, and 124.02 ± 256.74 pg/ml; for HC, class I-II, and class III-IV LN, respectively) (55). Another study observed that serum levels of IL-17A were significantly elevated in proliferative forms compared to non-proliferative LN (56).

### Requirement for Pulse Steroids and Response to Treatment

A study found that the presence of IL-17 in renal tissue correlated with the requirement for pulse steroids (*p* < 0.05) (49). In relation to response to treatment, in a study involving 52 patients with active LN (who underwent kidney biopsy at baseline and after immunosuppressive therapy), higher IL-17 levels at baseline were associated with persisting active nephritis after treatment (WHO III, IV, V) (42). At follow-up, non-responders had higher IL-17 (and IL-23) expression by inflammatory cells infiltrating renal tissue than responders (42). On the other hand, IL-17 and IL-23 decreased significantly in patients with active LN after 6 months of therapy (*P* < 0.001) (45). Another study showed that despite a progressive decrease in serum concentrations of IL-17A and IL-21 during induction therapy, the concentration of these cytokines remained higher in the non-remission than in the remission group (50).

### Renal Function, ESRD, and Mortality

Th17 cell frequencies significantly correlated with serum creatinine (53) and IL-17 was an independent risk factor for poor prognosis of LN (48). In another study, IL-17 immunostaining in biopsy correlated negatively with GFR (49). Table 1 summarizes the clinical studies that have assessed the role of the Th17/IL-17 axis in lupus nephritis.

**Table 1:** Clinical Studies on the role of the Th17/IL-17 axis and associated Imbalances in lupus nephritis.

**References**	**Year**	**Biomarker**	**Patients**	**Main results**
Xing et al. (57)	2012	Th17, IL-17, and IL-22	60 SLE patients and 28 healthy controls (HCs)	Patients with LN had a significant increase in the frequency of Th17 cells in peripheral blood, accompanied by FoxP3+ Treg cells decrease. So, the Th17/Treg ratio was significantly increased along with increased SLEDAI scores. The expression of IL-17 levels in LN patients exhibited a significant increase compared with patients without nephritis and healthy controls.
Dong et al. (58)	2003	IL-17	50 consecutively hospitalized LN patients and 15 adults HC who underwent blood samples to analyze the roles of IL-17 stimulation on the autoantibody's overproduction and IL-6 overexpression in PBMC.	In LN patients, the levels of IgG, anti-dsDNA, and IL-6 were higher in PBMC supernatants under IL-17 stimulation than in a normal culture medium. The increase in IgG, anti-dsDNA, and IL-6 levels, induced by IL-17, was dose-dependent and could be completely blocked by IL-17 monoclonal antibody and partially blocked by dexamethasone. During stimulation with IL-17, IL-6 mRNA levels were higher in LN patients than in HC (mean ± SD: 3.21 ± 0.24 vs. 1.30 ± 0.14, *P* < 0.05).
Cavalcanti et al. (59)	2017	IL-17 and IL-6	51 childhood-onset SLE patients (11 with LN) and 47 HC.	The levels of serum cytokines were significantly higher during active than inactive disease (mean ± SD: 6.14 ± 6.70 vs. 0.46 ± 1.47 pg/ml; P=0.041; and 13.64 ± 17.13 vs. 1.33 ± 0.86 pg/ml, *P* = 0.02; for IL-17, and IL-6 respectively).
Chen et al. (43)	2012	Th17 Cells, Serum and Glomerular IL-17 and IL-23 expression	24 LN patients (17 with class IV and 7 with class V), 12 HC, and 4 patients with MCD	The median frequency of circulating Th17 cells was significantly higher in LN patients than in HC [median (IQR): 0.68% (0.39–1.10%) vs. 0.12% (0.05–0.18%), *p* < 0.001]. Serum cytokine levels were significantly higher in LN patients than in HC (median: 7.26 vs. 0.82; 232.60 vs. 34.60; and 37.01 vs. 7.42 pg/ml, for IL-17, IL-6, and IL-23, respectively). The frequencies of circulating Th17-cells correlated positively with poor prognostic factors (SLEDAI, renal SLEDAI, histological activity index, cellular crescent, and endocapillary proliferation). Intraglomerular levels of IL-17 and IL-23 were significantly higher in class IV LN than in MCN patients or HC. Glomerular IL-17 and IL-23 expression levels were positively correlated with renal SLEDAI and histological activity index for LN patients.
Galil et al. (52)	2015	IL-17 and IL-6	72 SLE patients (30 with recent-onset active LN and 42 without renal disease) and 70 sex- and age-matched HC.	SLE patients were found to have significantly higher levels of IL-17 (*p* < 0.001) and IL-6 (*p* < 0.001) in relation to HC. Patients with LN had lower levels of both cytokines during periods of remission than in active disease (mean ± SD: 10.78 ± 2.38 vs. 19.54 ± 7.41 and 13.18 ± 2.73 vs. 28.46 ± 8.16, for IL-6 and IL-17, respectively, *P* < 0.001 for all). Elevated serum levels of both cytokines were associated with active LN, and anemia, and positively correlated with SLEDAI-2k scores (*P* = 0.025 for IL-17, and *P* < 0.001 for IL-6). There was a significant positive correlation between IL-6 and IL-17 serum concentrations during disease activity (*r* = 0.497, *P* = 0.005), as well as periods of remission of LN (*r* = 0.662, *P* < 0.001).
Zickert et al. (42)	2015	IL-17, IL-23, and other cytokines	52 patients with active LN who underwent kidney biopsy at baseline and after immunosuppressive treatment and 13 HC.	Baseline levels of IL-6, IL-10, IL-17, IL-23 were increased in patients vs. controls (*p* < 0.001 for all), as was IFN-γ (*p* = 0.03). Patients with persisting active nephritis after treatment (WHO III, IV, V) presented higher IL-17 levels at baseline than those who progressed without active nephritis (WHO I-II) (*p* < 0.03). At follow-up, BILAG-non-responders had higher IL-23 than responders (*p* < 0.05). This indicates that a subset of LN-patients has a Th17 phenotype that may influence response to treatment. Immunostaining of renal tissue revealed IL-17 expression in inflammatory infiltrates.
Susianti et al. (55)	2015	Urinary IL-17 (uIL-17)	50 participants with LN (38 with class III-IV, and 12 with class I-II) and 20 HC	The level of uIL-17 was significantly higher in the severe LN group than in the control group (*P* < 0.05); and increased with disease severity (mean ± SD: 43.96 ± 24.04, 55.69 ± 33.21, and 124.02 ± 256.74 pg/ml; for HC, class I-II, and class III-IV, respectively).
Yazici et al. (49)	2014	IL-17 and FOXP3	Renal tissue samples of 39 LN patients, and normal renal tissue as control (from 20 patients with Wilms' tumor who underwent nephrectomy).	Both IFN-γ (+) and IL-17+ cells were statistically higher in LN tissues when compared with controls (*p* < 0.01). The cells in the tubulointerstitium were CD3 + CD4+, displaying a Th1 and Th17 phenotype. IL-17 immunostaining correlated with proteinuria, the requirement for pulse steroids, and SLEDAI renal score; and correlated negatively with GFR. Furthermore, glomerular and interstitial IL-17 and IFN-γ stainings were significantly associated with various parameters of histological activity (*p* < 0.05).
Kshirsagar et al. (60)	2014	Peripheral Th17 cells, IL-17, and STAT3.	17 pediatric patients with LN, 5 patients with NS, and 24 age-matched HC	Compared to controls, LN children had a higher frequency of effector IL-17 producing cells in PBMCs, added to enhanced activity of Stat3 in these cells. The mRNA expression of IL-17 and retinoic acid-related orphan receptors was also higher in LN children than in controls.Additionally, Th17 cells from children with LN exhibit enhanced migratory capacity through high Akt activity.
Sigdel et al. (56)	2016	IL-A7, Th17 cells; and Th1 cytokines	49 patients with newly diagnosed LN (12 with LN-III; 32 with LN-IV; and 5 LN-V) and 24 HC.	Serum levels of IL-17A were significantly elevated in class IV LN compared to LN-V (*p* = 0.003) or HC (*p* = 0.001). IL-6 was increased in LN-IV when compared to LN-III and HC. Th1 cytokines (IFN-γ, IL-18) were also considerably expressed in LN IV patients' serum compared to HC. Additionally, the Th17/Th2 cell cytokines IL-17A/IL-4 ratio was significantly higher in LN-IV when compared with LN-III (*p* = 0.04), LN-V (*p* = 0.01), and HC (*p* < 0.0001).
Peliçari et al. (61)	2015	IL-17 levels	67 consecutive childhood-onset SLE patients, 55 first-degree relatives, and 47 age- and sex-matched healthy controls.	The serum IL-17 level was significantly higher in SLE patients than in HC [median (IQR): 36.3 (17.36–105.92) vs. 29.47 (15.16–62.17) pg/mL, *p* = 0.009]. There was an association between serum IL-17 levels and active nephritis (*p* = 0.01). Serum IL-17 levels were not associated with disease activity (*p* = 0.32), cumulative damage (*p* = 0.34), or medication use (*p* = 0.63).
Saber et al. (53)	2017	Peripheral Th17 cells and urinary IL-17	45 patients with SLE and 20 matching HC.	Th17 frequency and urinary level of IL-17 were significantly higher in patients than controls. Th17 cell frequencies and uIL-17 levels significantly correlated with renal biopsy classification for LN. Th17 cell frequencies significantly correlated with serum creatinine and SLEDAI; and inversely with C3 (*p* = 0.003), while uIL-17 significantly correlated with proteinuria and erythrocyte sedimentation rate.
AlFadhli et al. (62)	2016	Th17-related genes, IL-17A, and IL-17F	66 SLE patients (14 with LN) and 30 matched HC	Patients with LN had significantly higher serum concentrations of IL-17A (*P* = 0.002) and IL-17F (*P* = 0.002) than those without LN.Compared to HC, patients with SLE presented a difference in the expression of 14 Th17- related genes, including IL-17A and IL-17F.
Jakiela et al. (63)	2018	Th17 and Treg	33 LN patients and 19 HC.	The percentage of circulating Th17 among CD4+ cells was increased in LN compared to HC [median (IQR): = 1.2 (0.5–1.8) vs. 0.6% (0.32–0.95), *P* < 0.01]; without significant difference on Treg. Th17 expansion in the patient group was associated with a higher cumulative dose of cyclophosphamide but was not related to LN activity, renal histology, or blood and urine inflammatory biomarkers.
Wang et al. (50)	2018	Th17 cytokines (IL-17A and IL-21)	28 LN patients on induction therapy were assessed for serological data at weeks 0, 12, and 24.	There was a progressive decrease in serum concentrations of IL-17A and IL-21 (*P* < 0.01, *P* = 0.001, respectively) during induction therapy. The concentration of these cytokines remained higher in the non-remission than in the remission group. Additionally, the concentration of these cytokines at the baseline kept a positive correlation with the severity of proteinuria, ESR, SLEDAI scores, and ANA titers.
Edelbauer et al. (64)	2012	Th17, IL-17, and IL-23	23 patients with definite LN, 12 patients with frequently relapsing NS, and 20 age-matched HC.	There was a significant expansion of Th17 and Th1/Th17 cells in children with LN greater than in HC. Serum IL-17 and IL-23 levels correlated positively with the renal SLEDAI (*r* = 0.5516, *p* = 0.0029, and *r* = 0.6116, *p* = 0.0007, respectively).
Cheng et al. (48)	2019	IL-17	45 LN patients and 50 HC.	The IL-17 serum levels were significantly higher in LN patients than in the control group (*P* < 0.001). Serum IL-17 in LN patients was positively correlated with urinary protein (*r*= 0.436, *P* < 0.05). IL-17 was an independent risk factor for poor prognosis of LN (*P* < 0.05)
Dedong et al. (45)	2019	IL-17 and IL-23	80 patients with LN (37 of them accepted immunosuppressive therapy and followed up for 6 months) and 20 HC who underwent blood samples to analyze the roles of IL-17 and IL-23 in monitoring activity and predicting response to treatment in LN.	Baseline IL-17 and IL-23 were higher in patients with active LN than in those with inactive LN or controls (*P* < 0.001). IL-17 kept an inverse correlation with C3 (*r* = −0.44, *P* < 0.001). IL-17 and IL-23 decreased significantly in active LN patients after 6 months of therapy (*P* < 0.001). The baseline level of IL-23 was a predictor of response to the immunosuppressive treatment in patients with active LN, being lower in the complete response than in the partial response group (*P* = 0.0015) or non-response group (*P* = 0.013). IL-17 and IL-23 correlated with SLEDAI (*P* < 0.001).
Nakhjavani et al. (54)	2019	Serum IL-17 and TWEAK	50 lupus patients (25 with LN and 25 without) and 39 HC, who underwent blood samples to evaluate serum IL-17 and TWEAK as biomarkers to detect renal damage.	Increased levels of IL-17 and sTWEAK were observed in SLE patients compared to HC, and in LN compared to non-LN groups. There was a significant positive association between serum IL-17 and TWEAK levels and SLEDAI, proteinuria, nephritis activity index, and other clinical manifestations (*P* < 0.05).
Elkoumi et al. (65)	2012	IL-17A gene polymorphisms for three SNPs (rs2275913, rs8193036, and rs3748067).	320 Egyptian children and adolescents, diagnosed with JSLE (217 with and 103 without LN) and 320 matched HC.	The SNPs of IL-17 rs2275913 were significantly more frequent among JSLE patients than HC (21 vs. 7%, OR: 3.8; and 37 vs. 29%, OR: 1.4, for A/A genotype and A allele, respectively; *p* < 0.003 for both). No significant difference was found for other SNPs. Patients carrying the IL-17 SNPs rs2275913 were more likely to develop LN (OR: 5.64 and OR = 2.73, for A/A genotype and A allele, respectively).
Rastin et al. (66)	2016	IL-17, IL-6, IFN-γ, and Foxp3 genes.	20 patients with LN class IV, 20 sex- and age-matched SLE patients without LN as control who underwent blood samples.	The levels of IL-6, IL-17, IFN-γ, were significantly increased in patients with LN class IV than in those SLE patients without LN. The expression of Foxp3 genes was also significantly increased among class IV LN compared to those without; however, no significant difference was found in TGF-β expression between groups, suggesting the insufficient capacity of Treg to control the pathogenic role of IL-17-producing cells.

*BILAG, British Isles Lupus Assessment Group; ESR, erythrocyte sedimentation rate; HC, healthy controls; IHC, Immunohistochemistry; IQR, interquartile range; JSLE, juvenile systemic lupus erythematosus; LN, lupus nephritis; MCD, minimal change disease; NS, nephrotic syndrome; OR, odds ratio; PBMC, peripheral blood mononuclear cells; SLE, Systemic lupus erythematosus; SLEDAI, Systemic lupus erythematosus disease activity index; SNPs, single-nucleotide polymorphisms; STAT3, Signal transducer and activator of transcription 3; TWEAK, Tumor necrosis factor-like weak inducer of apoptosis; uIL-17, levels of urine interleukin-17; WHO, world health organization*.

## The Track and Footprints of Th17/IL-17 Axis Hyperactivity in Lupus

### From Extracellular Chromatin to APCs Maturation

An early event in the classical immunopathogenesis of SLE is the easy release of intracellular content into the extracellular space, the breakdown of immune tolerance to self, and autoantibodies production (4, 67). Components released into extracellular space function as danger-associated molecular patterns (DAMPs) (4, 68) and are recognized by dendritic cells, and other antigen-presenting cells (APCs), through toll-like receptors (TLR4) present in their plasma membrane (69–72). On the other hand, autoreactive B cells respond to immunogenic DNA with autoantibodies production (67, 71, 73), which APCs also internalizes (through FCγRII) as DNA-containing immune complexes (68) and then recognized by TLR7 and TLR9 present in endosome (69, 74, 75). The binding of DAMPs to TLRs in APCs induces their maturation (76, 77). Mature APCs, in turn, drive lymphocyte activation (78).

### Activation and Differentiation of Th17 Cells

Th17 cells differentiate from naive T auxiliary cells, according to microenvironmental factors, in the presence of IL-1β, IL-6, IL-23, and TGF-β, which are the key cytokines for its differentiation (78–80) and requires the lineage-specific transcription factor retinoid-related orphan receptor-gamma (RORγt) (80, 81). As described above, Mature APCs (after the binding of DAMPs to TLRs) trigger lymphocyte activation by the interaction of MHC II with TCR and several co-stimulatory molecules (15, 78, 80, 82). In the context of this interaction, mature APCs produce the key cytokines for Th17 differentiation (78, 80), using nuclear factor kappa B (NF-κB) and/or mitogen-activated protein kinase (MAPK) as signaling pathways (72, 77, 83). These cytokines bind to their respective receptors in naive CD4+ T Cells and trigger a chain of events downstream involving the Signal transducer and activator of transcription 3 (STAT3), which stimulates the synthesis of IL-17 and IL-21 either by binding directly to their genes or by activating RORγt (80, 84). Interestingly, T cells from SLE patients presented enhanced Stat3 activity added to higher RORγt expression (85). Once differentiated, Th17 cells secrete their cytokines (IL-17A, IL-17F, IL-17C, IL-21, and IL-22), most of them with a pathogenic role in the kidney (32, 86–88), in addition to its systemic effects (1, 15).

Regarding Th17 cells differentiation, it is also worth mentioning that podocytes, mesangial cells, and renal tubular epithelial cells can behave as antigen-presenting cells (89–92). So, these intrinsic renal cells can alone trigger the local activation of Th17 cells after recognizing, processing, presenting eventual DAMPs that cross the glomerular filtration barrier, as seen in other kidney disease models (89, 93, 94). Interestingly, a study showed that IL-17 (and IFNγ) upregulated the expression of MHC-I, MHC-II, and co-stimulatory molecules (CD80 and CD86) on the podocyte surface. Moreover, under IL-17 stimulation, podocytes increased the uptake and processing of antigen, resulting in the presentation of its peptide on the cell surface (93). This fact brings robustness to the idea that, in part, naïve T cells can enter the kidney and continue, under local factors, in the path of differentiation and activation to Th17 (95, 96). A recent study reinforces this thesis by demonstrating that, in kidneys of patients with ANCA-associated glomerulonephritis, Th17 cells develop from CD4+ tissue-resident memory T cells and exacerbate renal pathology by secreting IL-17A (97, 98).

### Th17 Polarization in SLE and Related Immunes Imbalances

Although the same general mechanisms regulate the activation of all T-cell subtypes (effector and regulatory) it is worth emphasizing that in the lupus autoimmunity environment, there is favoritism of self-reactive effector (4, 73, 99); with the detriment of regulatory cells (17, 97, 100). One of the bases of this polarization lies in the fact that there are plasticity and reciprocity between Th17 and Treg (100–102), a balance influenced by various factors (103), and the inflammatory and autoimmunity environment of lupus favors to the side of the Th17 cells (2, 79, 101). Added to Th17/IL-17 axis overactivity, LN is characterized by decrease, suppression, or dysfunction of Treg cells (57, 104) and impairment of other protective factors like IL-2 (97) and IL-10 (105, 106).

Several aspects present in SLE favor the polarization of CD4+ cells to a proinflammatory profile (Th1 and Th17) (2, 107). This range from phenotypic and functional aberrations of APCs (2, 108, 109) to T cells specific aspects, like changes in immunometabolism (marked glycolysis, lipid synthesis, glutaminolysis, and hyperactivation of the mTOR pathway) (110, 111), and abnormalities in signaling pathways (112, 113). In addition, epigenetic changes, such as histone hypomethylation at naive CD4+ T Cells level, have also been described to favor Th17 polarization (114, 115) and were early events before lupus flares (114). Additionally, dysbiosis, a characteristic also present in lupus (116, 117), is a potentiating factor for Th17/IL-17 polarization (118); and a study has even shown that autoimmune kidney disease is exacerbated by the migration of pathogenic Th17 cells from the intestine to the kidney (119).

In the lupus autoimmunity environment, other elements of the immune response participate in Th17 polarization, as shown in an experimental study with lupus-prone mice, in which dendritic and B cells increased Th17 expansion, associated with limited Treg expansion, and increased renal infiltration by Th1 and Th17 cells (2). In another study, basophils obtained from patients with SLE promoted Th17 differentiation from SLE naïve CD4+ T cells *in vitro* coculture (120). Even cells with a dominant protective role, like Treg cells, in the lupus nephritis background, have been shown to facilitate the proliferation of Th17 lymphocytes and are less suppressive (47, 63). Several other elements of the immune response favor polarization to Th17 in the context of lupus (121).

## Mechanisms Underlying the Effects of Th17/IL-17 Axis in the Kidney

### Recruitment of Th17 Cells to the Kidney

Th17 cells are attracted to the kidney by chemokines CCL20, CXCL9, and CXCL10 (33, 122, 123) through binding to their receptors (CXCR3 and CCR6) expressed on the surface of these cells (124–126). Recruitment is facilitated by the enhanced migratory capacity of Th17 cells from SLE patients, through high Akt activity (60) and involvement of calcium/calmodulin-dependent kinase IV (CaMK4) (96, 127). Most intrinsic kidney cells (podocytes, mesangial, and kidney tubular cells) secrete CXCL9, CXCL10, and CCL20 in response to injury (23, 123, 128). In an experimental study, stimulation of mesangial cells with nucleosome-containing immune complexes resulted in their activation and expression of CCL20 (129). More recently, it has been shown that components of the extracellular matrix, produced by injured cells, stimulate resident macrophages to produce CCL20, CXCL9, and CXCL10, cooperating in this way in the recruitment of Th17 cells (130).

Once in the kidney, Th17 cells maintain the phenotypic and functional features through several other factors, as demonstrated in models where local T cells had elevated expression of inducible T cell costimulator (ICOS) coreceptor and were protected from apoptosis by elevating the activity of the PI3K-Akt signaling pathway. These features together result in facilitating the accumulation of active T cells in the kidney (131, 132). Additionally, a clinical study revealed that LES patients had elevated serum autoantibodies against co-inhibitory PD-1, facilitating T cell proliferation and maintaining the hyperactive phenotype; and this kept a close association with disease activity, particularly renal involvement (133).

### Effects of Th17/IL-17 Axis in the Kidney

In the kidney, the Th17/IL-17 axis participates in several points of the damage chain. In summary, this involves changes in the structure and functioning of intrinsic specialized renal cells, promoting and maintaining an inflammatory environment, participating in repetitive tissue damage and maladaptive repair, leading to renal fibrosis and loss of function (23, 25). Thus, the Th17/IL-17 axis behaves as a true chief orchestrator of immunity (134, 135). The available evidence on the Th17/IL-17 axis effects on specific renal cells or compartments is described below in this review.

#### Glomerular Compartment

Regarding the effect of the Th17/IL-17 axis on the filtration barrier elements, there is growing evidence about the harmful effect of IL-17 on cellular elements of this barrier; however, it remains a field in need of an extensive investigation.

##### Podocytes

On podocytes, an experimental study raised the possibility that Th17 cells would produce a soluble mediator that enhances podocyte motility, causing rearrangement of the actin cytoskeleton and increased permeability (136). This finding may be the basis of the correlation found between IL-17 levels and proteinuria and its severity (50, 53). According to the study, this soluble factor mimics the protease-activated receptors-1 (PAR-1) activation signaling pathways (136).

Still focusing on the potential impact on the cytoskeleton of podocytes, the exposure of mice podocytes to recombinant IL-17 induced overproduction of Cmaf-inducing protein(c-mip), with consequent induction of cytoskeletal disorganization and apoptosis in adriamycin-induced nephropathy model (137). Interestingly, silencing c-mip prevented IL-17 related podocyte apoptosis by promoting persistent activation of NF-κB and upregulation of anti-apoptotic protein Bcl-2 (137). C-mip is a protein whose expression is suppressed in healthy glomeruli (138) and increased in pathological conditions and has been associated with cytoskeletal disorganization in podocytes and proteinuria (139, 140).

In another experimental study with mouse podocytes, IL-17A stimulation disrupted the podocyte morphology by decreasing podocin expression and increasing desmin expression. In this study, podocytes expressed IL-17RA, and stimulation with IL-17A induced changes associated with activation of the NLRP3 inflammasome-caspase-1 pathway, production of intracellular reactive oxygen species (ROS), and increased IL-1β secretion. Interestingly, the blockade of these downstream signaling pathways restored the podocyte morphology (141).

Additional evidence about the harmful effect of IL-17 on podocytes comes from studies involving patients with primary nephrotic syndrome (PNS) (142). In these patients, IL-17 was highly expressed in renal tissue, being higher in patients with focal segmental glomerulosclerosis (FSGS), the glomerular disease with greater fibrosing behavior. As increased the expression of IL-17 Messenger RNA (mRNA) in the tissue, decreased the expression of podocalyxin (PCX) mRNA; and the IL-17 mRNA correlated directly with the number of podocytes lost in the urine. In the complement, with *in-vitro* experiment, IL-17 induced podocytes apoptosis and reduced podocyte health proteins such as nephrin, synaptopodin, and PCX. At the same time, IL-17 induced the expression of proteins like Fas, Fas ligand (FasL), active-caspase-3, active-caspase-8, and phosphorylated-p65. These effects occurred with the involvement of NF-κB pathways, and its inhibition attenuated the IL-17-induced podocyte apoptosis, decreasing or suppressing the molecular pathways described above (142). In another study, exposure of murine podocytes to recombinant IL-17 also induced apoptosis, increased the expression of caspase-3, caspase-8, and Fas; associated with decreased PCX expression, in a dose- and time-dependent manner (143).

##### Mesangial Cells

In mesangial cells, stimulation with IL-17A or IL-17F induces the production and release of chemokines CCL2 and CXCL2 in a MAPK-dependent manner. Both IL-17RA and IL-17RC are expressed in these cells, and the production of the chemokines was in a dose- and time-dependent manner (32).

##### Glomerular Endothelial Cells

Concerning glomerular endothelial cells (GEC), there are no specific studies in these cells. What is known are the effects of the axis on the endothelium from other vascular beds (see description in the section hypertension and thrombosis). However, these effects we believe to be applicable (in whole or part) to GEC. Specific studies are needed to assess potential local-specific effects.

##### Glomerular Basement Membrane

Although little is known about the potential effect of IL-17 on glomerular basement membrane (GBM), the IL 17 presence was associated with GBM thickening in a model of accelerated diabetic nephropathy; while IL-17A blockade with antibody reduced this effect (144). Additionally, in a model of anti-glomerular basement membrane glomerulonephritis (anti-GBM GN), the Th17/IL-17 pathways were drivers of inflammation and autoantibody-induced renal injury; and the knockout or inhibition of IL-17 ameliorated these effects associated with decreased proinflammatory cytokines (145, 146).

#### Tubulointerstitial Compartment: Tubular Epithelial Cells, Fibrosis, and Epithelial-Mesenchymal Transition

##### Renal Tubular Epithelial Cells and Inflammation

The Exposure of RTEC to IL-17 induces the production of various mediators, from cytokines, chemokines, and growth factors as shown in several studies and experimental models, and both receptors (IL-17RA and IL-17RC) are expressed in these cells. Stimulation of RTEC with IL-17 increases the expression of various cytokines like IL-6, IL-1β, TNF-α (31, 147).

In a model of crescent glomerulonephritis by lupus, RTEC stimulated with IL-17 and IFN-α significantly increased the expression of CCL2, which is chemotactic for dendritic cells and macrophages. In mice lacking IL-17RA, renal infiltration by macrophages was severely impaired, despite unchanged systemic response (147). In addition, stimulation of tubular epithelial cells with IL-17 increased mRNA expression of other chemokines like Cxcl1, Cxcl2, and Cxcl8, which are chemotactic for monocytes and neutrophils, as found in models of autoimmune glomerulonephritis. These effects were synergically potentiated by TNF-α (148). Thus, under IL-17 stimulation, RTEC produces mediators that recruit dendritic cells and macrophages that are important sources of TGF-β to promote renal fibrosis (149) putting IL-17 as an important driver of RTEC-mediated immunopathogenesis in LN.

The stimulation of RTEC with IL-17A impacts neutrophil kinetics, leading to the synthesis of the granulocyte colony-stimulating factor (G-CSF) in a dose- and time-dependent manner and this effect occurred in synergy with TNF-α- or IL-1β. The downstream signaling pathways of this effect involved MAPK activation (31). Added to G-CSF secretion, the stimulation of RTEC with IL-17A induced the expression of chemokines CXCL1 and CXCL5 that are responsive for massive neutrophil recruitment and consequent renal tissue injury (86, 150). Taken together, IL-17 is a potent orchestrator of neutrophil-mediated damage, promoting both differentiation and the recruitment of neutrophils to the kidney (31, 150).

##### IL-17, Renal Tubular Epithelial Cells, and Fibrosis

The Th17/IL-17 axis is a potent promoter of renal fibrosis (39, 151) as found in an experimental model of unilateral ureter obstruction (UUO), where TGF-β1 expression (mRNA and protein) were increased in the obstructed kidney (39). In the complement of the study, the addition of IL-17A to cultured renal proximal tubular epithelial cells or renal fibroblasts increased the production of fibronectin using the TGF-β/Smad signaling pathway; associated with increased expression of TGF-β1 mRNA and protein (39). Interestingly, the IL-17A-mediated fibronectin production was abrogated neutralizing TGF-β1 pathways, either by administering an anti-TGF-β1 antibody or TGF-β1 receptor I inhibitor (39).

In another study, IL-17A promoted myofibroblast activation and extracellular matrix deposition, and IL-17 deficient mice were protected from fibrosis secondary to obstruction (36). In an experimental model of hypertension and angiotensin II-induced fibrosis, the IL-17A or IL-17RA blockade with specific antibodies significantly reduced the fibrosis marker TGF-β1 (152). On the other hand, the antifibrotic effect of many agents in the kidney has been associated with reducing IL-17 (153–156).

##### IL-17 and Epithelial-Mesenchymal Transition

Some scant literature shows that the Th17/IL-17 axis induces Epithelial-Mesenchymal Transition (EMT) on tubular epithelial cells (157, 158). In one of these studies, with cultured cells, IL-17A promoted the cellular proliferation and secretion of extracellular matrix and induced inversion from epithelial to mesenchymal phenotype in a TGF-β1-dependent pathway (157). Despite few studies in the kidney, the effect of the axis on EMT is well-known in many organs such as bronchoalveolar epithelium (159, 160); epithelial cells of the salivary glands in Sjögren's syndrome (161, 162); biliary epithelial cells (163, 164); and peritoneal mesothelial cells (165). Concerning the promotion of the same effect on other intrinsic cells (mesangial and glomerular endothelial cells), it is an open gap to be elucidated in future investigations.

#### Vascular Compartment: Thrombotic Microangiopathy and Hypertension

One of the factors of poor prognosis in renal biopsy in lupus is thrombotic microangiopathy (which is the combination of endothelial injury and thrombosis). No primary studies evaluated the role of the Th17/IL-17 axis in thrombotic events in patients with lupus. However, IL-17A, IL-17RA, or IL-23 probably participate in this process because they are described as mediators of endothelial dysfunction (166, 167); and have been associated with the occurrence of arterial thrombosis (167, 168). In experimental studies, with psoriasis models, IL-17A shown to be a mediator of thrombotic events and vascular dysfunction (169, 170). In another study with endothelial cells from patients with rheumatoid arthritis (RA), IL-17 (in combination with TNF-α) induced a procoagulant and prothrombotic phenotype (beyond the inflammatory state). Mechanistically, this occurred due to the strong inhibition of the expression of CD39/ATPDase (an inhibitor of platelet activation), enhancement of tissue factor (the cellular receptor for FVII and FVII), combined to decreased thrombomodulin (167). Additionally, studies have found an increase in Th17/IL-17 axis activity in primary antiphospholipid syndrome (171, 172).

Hypertension is one of the manifestations of kidney involvement in lupus, and its presence is one of the factors of poor prognosis in LN (173, 174). There is a lack of primary studies evaluating the direct effect of the Th17/IL-17 axis in this event in LN. However, there is evidence associating the Th17/IL-17 axis with primary hypertension and renal inflammation in both experimental and human studies (175, 176). Additionally, basic research with angiotensin II-induced hypertension models shows that IL-17A deficiency or the blockade of IL-17A or IL-17RA with specific antibodies significantly reduces the pressure and inflammation in target organs (152, 177). In another experimental study, IL-17A appeared to be a key mediator of vascular remodeling of the small arteries. Increased IL-17A levels increased blood pressure by induction of arterial remodeling and stiffness. In addition, treatment with antihypertensive drugs lowered blood pressure without modifying structural changes. Conversely, blocking the IL-17A with antibodies decreased blood pressure and vascular remodeling, suggesting that it has a sustained effect on vascular structure, more than merely hemodynamic (178). So, despite the lack of primary studies focusing IL-17 and hypertension on NL, it is believed that there is a participation of the axis in this outcome since lupus is a disease that occurs with significant hyperactivity of the Th17/IL-17 axis.

#### The Th17/ IL-17 Axis and Local Immune Response Orchestration

##### Chemokines Production, Recruitment of More Immune Cells, Tertiary Lymphoid Structures Formation

As already described, the Th17/IL-17 axis induces the expression of chemokines like CXCL5, CXCL2, and CXCL8 for the recruitment of neutrophils (by binding to the receptors CXCR1 and CXCR2) (23, 32, 150), CCL2, CCL5 to attract monocytes and macrophages (by binding to receptors CCR1, CCR2, CCR5) (32, 36). In addition to IL-17-induced chemokine production, several other chemokines like CXCL13 (179, 180), CCL2, CCL7, CXCL1, CXCL2, and CXCL5 are produced by injured cells and resident macrophages, promoting the infiltration of B cells, dendritic cells, NK, Th1 cells (181–183) increasing the recruitment of more immune cells to the organ (184). The Sustained recruitment of immune cells can lead to the formation of kidney tertiary lymphoid structures (TLS), with some autonomy, in local activation of effector T cells, and *in situ* production of autoantibodies and components of the complement system (185, 186), sustaining by itself the inflammatory flame at the local level (95, 187, 188). A recent experimental study showed that IL-17A is an orchestrator of TLS formation in the kidney, and this formation is associated with intrarenal inflammation, fibrosis, and progression of kidney damage (189). Interestingly, genetic depletion of IL-17A or blockade with anti-IL-17A antibody significantly reduced TLS formation, associated with attenuation of renal inflammation and fibrosis (189).

##### Production of Autoantibodies in situ, Complement Activation, Immunocomplex Formation, and Tissue Deposition

The IL-17 seems to participate in the production of autoantibodies, *in situ*, probably involving tertiary lymphoid structures, as found the correlation between IL-17 and increased anti-double-stranded DNA (dsDNA) production in an experimental study with kidney biopsy (185). IL-17 also seems to participate in other *in situ* events, including complement activation, immunocomplex formation, and tissue deposition, as found in the association of its expression level with these critical events in LN (185). In another study, it was evidenced that in IL-17A–/– mice, there was a decreased glomerular IgG and complement deposition and decreased intrarenal expression of Th1-associated proinflammatory mediators (190).

##### Crosstalk Between Th17; Intrinsic Renal Cells and Resident Immune Cells in Kidney Diseases

There is a Crosstalk between the kidney cells and Th17/IL-17 axis since the intrinsic cells of the kidney can induce the polarization of the lymphocytic response to the Th17 profile; as shown in an experimental study, in which stimulation of podocytes with bacterial products Polarized Naive CD4+ T Cells into Th17 cells (94). In addition, there is a crosstalk between Th17/IL-17 and intrarenal immune cells as shown that resident dendritic cells and infiltrating monocytes secret IL-1β that activated intrarenal Th17 cells and enhanced the IL-17 secretion (95). Elevated levels of IL-17, in turn, stimulate intrinsic kidney cells to produce chemokines and G-CSF/GM-CSF, inducing the differentiation of neutrophils and macrophages from bone marrow and recruitment to the kidney (31, 191, 192). Together, these aspects show that IL-17 participates in the cross-talk between Th17, neutrophils, monocytes, and intrinsic kidney cells (32).

**Figure 1:**
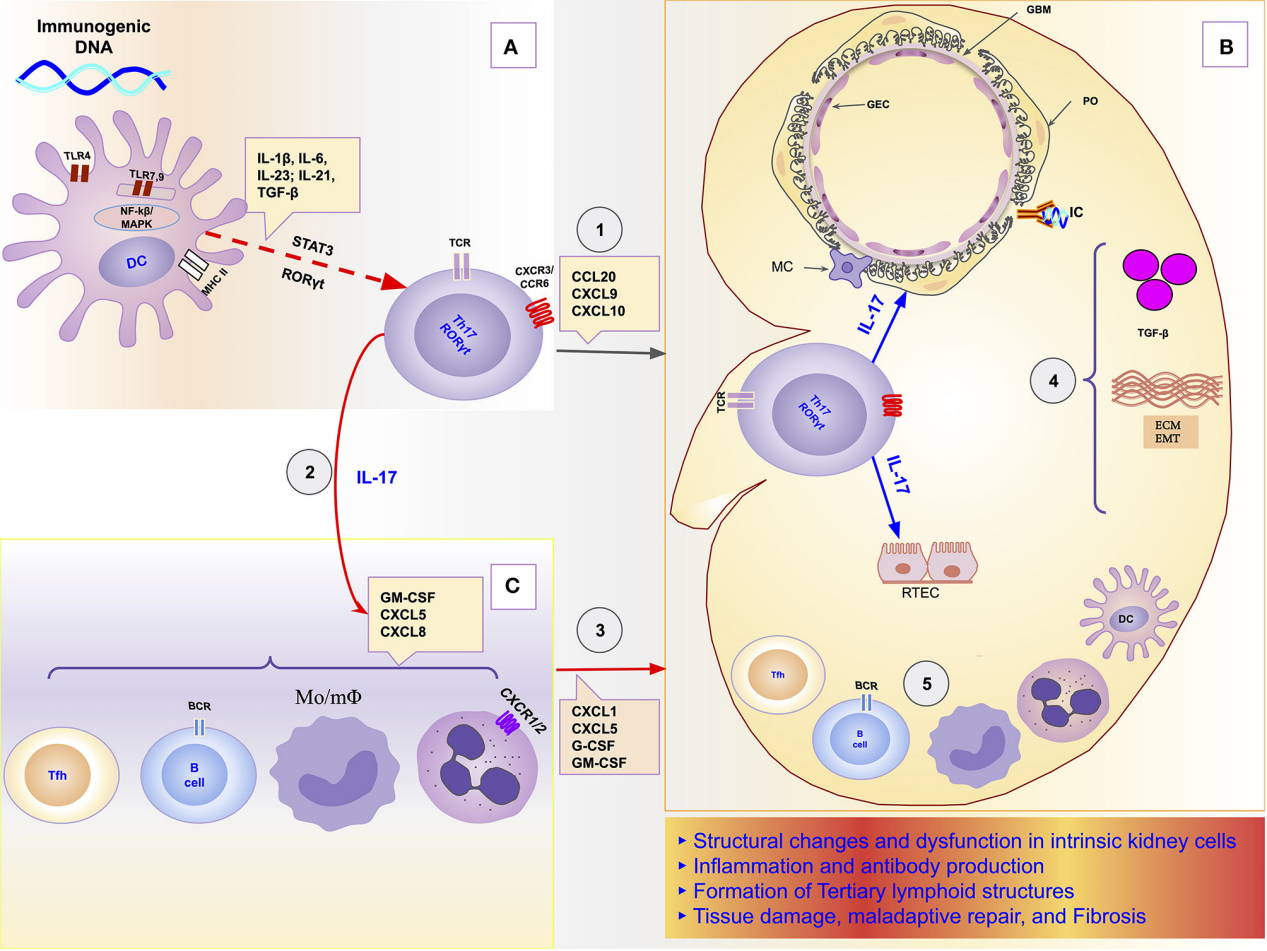
Schematic representation of the role of the Th17/IL-17 axis in the chain of events to kidney damage and ESRD in Lupus Nephritis. **(A)** Dendritic cells sense extracellular DNA through TLR4 present in their plasma membrane (69, 71, 72) or sense phagocyted DNA-containing immune complexes by TLR7 and TLR9 in endosome (69, 74, 75). The binding of DAMPs to TLRs in APCs induces their maturation (76, 77). Part of dendritic cells migrates to draining lymph nodes to present the processed antigen to T cells and induce their activation and differentiation. The differentiation of Th17 cells is promoted by proinflammatories cytokines (IL-1β, IL-6, IL-21, IL-23) that dendritic cells secrete using the NF-κB and MAPK signaling pathways (77, 83). Dendritic cells that remained in the tissue secrete various chemokines like CXCL9, CXCL10, and CCL20 that drive the recruitment of Th17 cells to the kidney through binding to receptors (CXCR3 and CCR6) (96, 125, 126). **(B)** In the Kidney, Th17 releases its cytokines (IL-17A, IL-17F, IL-17C, IL-21, and IL-22) that act directly on intrinsic kidney cells (mesangial cells, podocytes; glomerular endothelial cells, renal tubular epithelial cells). IL-17 family cytokines are responsible for changes in the cytoskeleton of the podocytes, activation of inflammasome and caspases, and induction of oxidative stress and podocytes apoptosis. In addition, in tubular epithelial cells, IL-17 promotes the activation of the profibrotic pathways with the increase of the expression of TGF-β (36, 87), promotion of EMT (158) with consequent increase of extracellular matrix proteins and fibrosis (87). **(C)** Besides local effects, IL-17 amplifies the systemic inflammatory response by stimulating the synthesis of inflammatory cytokines, growth factors, and chemokines, resulting in granulopoiesis/myelopoiesis and recruitment of more immune cells to the kidney (31, 32, 194). In addition, it promotes autoantibody production by its effects on Tfh and GC (195, 196), and plasma cells (197). DC, dendritic cells; ECM, extracellular matrix; EMT, epithelial-mesenchymal transition; GBM, glomerular basement membrane; GEC, glomerular endothelial cells; IC, immune complex; IL-1, Interleukin-1; IL-17, Interleukin-17; IL-21, Interleukin-21; IL-23, Interleukin-23; IL-6, Interleukin-6; MAPK, Mitogen-activated protein kinase; MC, mesangial cell; *Mo*/*m*Φ, Monocytes/macrophages; NF-κB, Nuclear factor-κ B; PO, podocyte; RTEC, renal tubular epithelial cells; Tfh, follicular helper T cells; TGF-β, transforming growth factor-beta; Th17, T helper lymphocytes, subtype 17; TLR2, Toll-like receptor 2; TLR4, Toll-like receptor 4.

In this orchestrator role of local immune response, it was demonstrated, in a model of obstructive nephropathy, that monocytes and macrophages express the IL-17RA receptor, and the absence of this receptor in all myeloid cells resulted in a reduction in macrophage accumulation in the kidney and significant attenuation of fibrosis (192). IL-17 participates as a mediator or potentiator of renal damage caused by several other cells and molecules of the immune response. For example, in a model of obstructive nephropathy, the C3 component produced locally by macrophages promoted renal fibrosis through increasing T-cell proliferation and IL-17A expression. Furthermore, the blockade of C3a reduced IL-17A expression and tubulointerstitial fibrosis (38). The Th17/IL-17 axis even seems to be able to initiate the chain of kidney damage by itself as it possesses the property of activating the inflammasomes and the toll-like receptors (193). Figure 1 is a schematic representation of the chain of events from hyperreactivity to self-DNA, activation and polarization of the Th17/IL-17 axis, to kidney damage and ESRD.

### The Association Between Th17/IL-17 Axis Hyperactivity and Several Kidney Diseases (Other Than Lupus Nephritis)

The Th17/IL-17 axis role as a mediator of kidney damage and fibrosis has been found in various other renal diseases (in both patients and animal models) (198–200). These include primary glomerular diseases (198, 201), diabetic nephropathy (199, 202, 203), hypertensive nephropathy (175) ischemia-reperfusion models (37, 87, 200), renin-angiotensin-aldosterone system-mediated damage (204, 205), unilateral ureteral obstruction associated damage (36, 95, 206), and in ablation or unilateral nephrectomy associated damage (207).

Th17/IL-17 axis seems to increase the risk of CKD itself, as seen in a genetic study with 650 elderly, where single nucleotide polymorphism (SNP) of IL17RA (rs4819554 AA homozygotes) was significantly more frequent among individuals with eGFR < 60 ml/min/1.73 m^2^; and was associated to the risk of developing ESRD (40). Another study including 290 non-diabetic ESRD patients and 289 normal controls found that patients had a significantly higher frequency of IL17E rs10137082^*^C and IL17RA rs4819554^*^A alleles compared to control. At the same time, the genotyping analysis found that SNPs for IL17E (rs10137082) and IL17RA (rs4819554) were significantly more frequent among patients than in controls, after adjusting for confounders (208). It is important to highlight that this is an association and not necessarily a causal relationship because SNPs are hardly a causal factor alone. This is even less likely in a disease like lupus, with heterogeneous and multifactorial etiology (5, 209). However, associated SNPs may be players with additive or synergistic effects at the confluence of the multi-players that characterize the disease (11, 111).

Additional evidence about the role of the Th17/IL-17 axis on Kidney diseases comes from the observation of the increased risk of CKD associated with renal inflammation in human diseases that occur with the hyperactivation of the Th17/IL-17 axis, like psoriasis (210–212), rheumatoid arthritis (213, 214), and ankylosing spondylitis (215–217). In two studies (in the United Kingdom and Taiwan), psoriasis was associated with an increased risk of chronic kidney disease independent of traditional risk factors (211, 212). The myriad of exposed situations suggests that Th17/IL-17 is a permanent participant, or at least as a pivotal element, in the pathogenesis of many kidney diseases independent of the initial insult (218); and its role extends from initial mechanisms, ESRD, to consequences of CKD and dialysis (88, 205, 207, 219).

**Figure 2:**
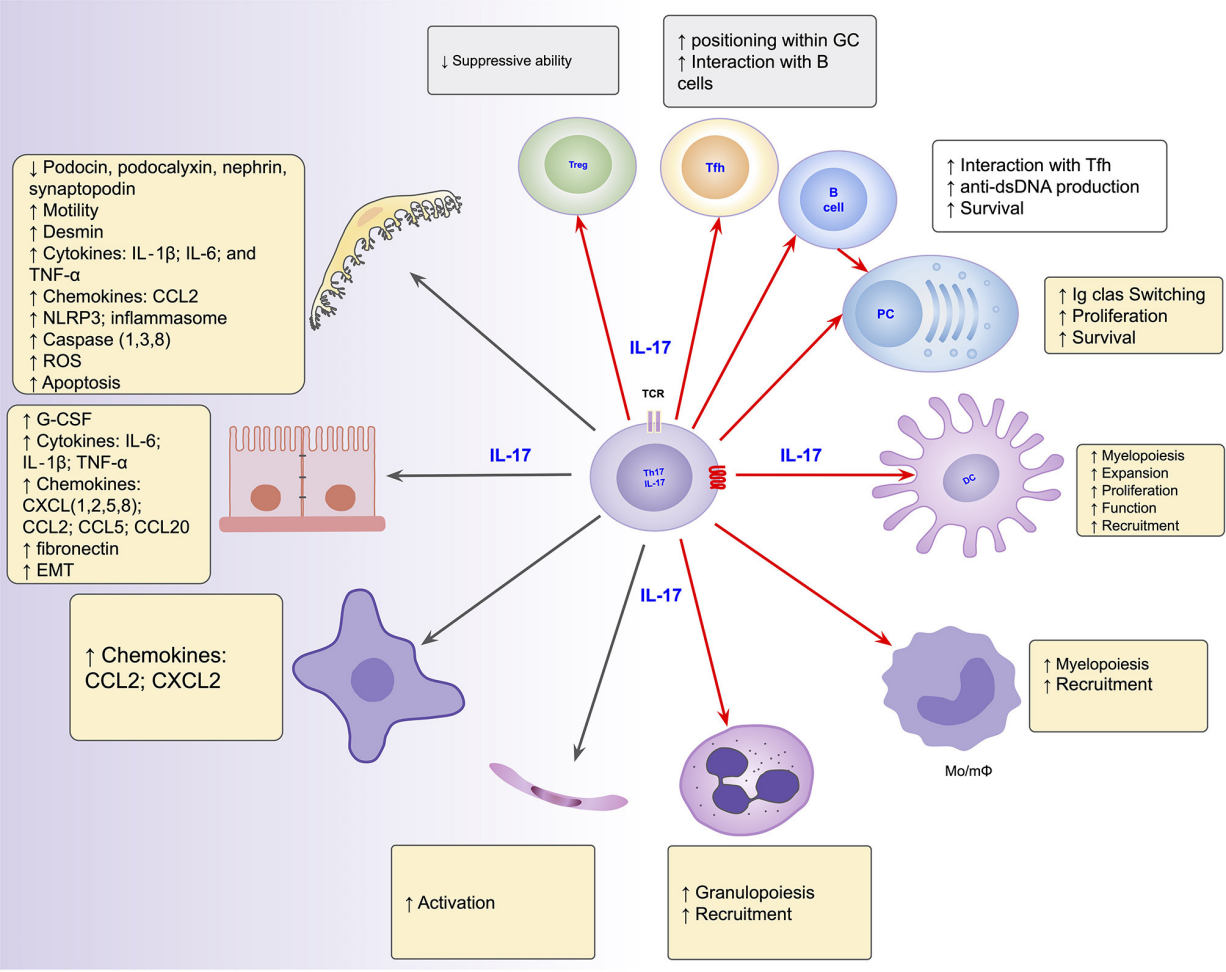
Summarized effects of the Th17/IL-17 axis on intrinsic renal cells (black arrows), as found in several kidney disease models, and on immune cells (red arrows) potentially implicated in the induction of kidney damage in lupus nephritis. DC, dendritic cells; EMT, epithelial-mesenchymal transition; IL-1, Interleukin-1; IL-17, Interleukin-17; IL-6, Interleukin-6; Mo/mΦ, Monocytes/macrophages; PC, Plasma Cells; Tfh, follicular helper T cells; Th17, T helper lymphocytes, subtype 17; Treg, Regulatory T Cell.

The role of the Th17/IL-17 axis has been found in other organic diseases that combine both inflammatory and fibrosing courses (219–221). Thus, the axis is involved in intestinal fibrosis in inflammatory bowel disease (222, 223), in pulmonary fibrosis in systemic sclerosis and cystic fibrosis (220, 224), liver cirrhosis (221, 225) and peritoneal fibrosis (219, 226). Its blockage and/or suppression has emerged as promising to prevent/mitigate the inflammatory and/or fibrosing behaviors in such conditions (226–228). Figure 2 summarizes the Th17/IL-17 axis effects on intrinsic renal cells and immune cells with potential implications in kidney damage, particularly in lupus nephritis.

## Systemic Effects of Th17/IL-17 Axis With Repercussions in the Kidney

### Production of Autoantibodies

The Th17/IL-17 axis participates in the production of autoantibodies by B cells, as demonstrated in studies with autoimmune models in which the IL-17 drives the development of autoreactive germinal center (GC); and mice lacking the IL-17 receptor have reduced B cell development and humoral responses (229, 230). In another study with an autoimmune disease model, the blockade of IL-17 signaling was associated with a significant reduction in both the number and size of germinal centers (231).

The IL-17RA receptor is essential for the optimal location of follicular helper T cells (Tfh) in the light zone (LZ) of the GC to promote the production of autoantibodies by B cells (195). Additionally, the production of IL-17 initially correlates with a reduced migratory response of B cells to chemotactic like CXCL12, suggesting that IL-17 not only facilitates the interaction between Tfh and responder B cells but also prolongs this interaction by increasing the time of permanence of B cells in GC (229).

In relation to the structure and functioning of the germinal center, a lymph node study showed that IL-17 is a critical requirement for the proliferation of lymph node and splenic stromal cells, particularly fibroblastic reticular cells (FRCs), during experimental autoimmunity. Without IL-17 signaling, there was a failure in FRC proliferation (nutrient stress, arrested cell cycle, and apoptosis), resulting in the impaired germinal center formation and antigen-specific antibody production (196).

The IL-17 importance in the production of autoantibodies was also evidenced in another study in which the PBMC supernatants from LN patients expressed higher levels of IgG, anti-dsDNA under IL-17 stimulation than in a normal culture medium. This effect occurred in a dose-dependent manner, and could be blocked completely by IL-17 monoclonal antibodies or partially by dexamethasone (58). Another experimental study showed that IL-17 increased anti-double-stranded DNA antibody production, and this was the link in the correlation between cytokine levels and disease severity (185). A recent study has ratified the crucial role of IL-17 by demonstrating that IL-17 promotes autoantibody production and increases plasma cell survival. In this study, the subset of plasmocytes expressing the IL-17RC receptor had an exponential increase in the production of anti-dsDNA IgG upon IL-17A stimulation in both patients and mice. Additionally, the transfer of Th17 depleted PBMC resulted in a significant reduction of autoantibody production and attenuation of renal damage. This attenuating effect was also observed in IL-17 or IL-17RC deficient mice, while the adoptive transfer of Th17 to IL-17-deficient mice restored the plasma cell response and renal lupus damage. The most important is that IL-17 significantly promoted plasma cell survival, through phosphorylation of p38, stabilizing Bcl2l1 mRNA, which encodes the anti-apoptotic protein Bcl-xL (197).

### Amplification of Systemic Inflammatory Response

Amplification of the inflammatory response is among the systemic effects of IL-17. This includes granulopoiesis and myelopoiesis by stimulating the synthesis of GM-CSF and G-CSF, increased production of chemokines, and inflammatory cytokines (31, 192); in addition to associated paralysis or impairment of anti-inflammatory pathways (105). In these effects, IL-17 makes synergy with several other inflammatory mediators such as IFN-γ, TNF-α, and IL-23 (42, 49, 232, 233). In a PBMC culture medium, stimulation with IL-17 induced significant IL-6 mRNA transcription in PBMC from LN patients than from HC (58). This study also brings the notion that, compared to controls, cells from lupus patients are hyperresponsive with higher production of inflammatory mediators under the same stimulus conditions (58).

In neutrophil kinetics, especially, IL-17 participates in various points of the chain, from differentiation through the synthesis of colony-stimulating factors (31); recruitment of neutrophils to the target organs through endothelial cell activation in a STAT3 and/or MAPK-dependent manner (34, 234) and by the synthesis of attracting chemokines like CXCL5, CXCL1; and CXCL8/IL-8 (86, 235). In relation to CXCL1, a potent chemoattractant for neutrophils, it is worth mentioning that IL-17 participates in regulating its production and in the stability of its mRNA (232, 235) and increases its biological half-life (236).

The Th17/IL-17 axis seems to have a cooperative relationship with other pathways whose importance is highlighted in the pathogenesis of SLE, like Type I interferons (6, 11, 237). This was evidenced by studies that found increased IL-17A and IL-17A-producing cells in IFN+ than in IFN- patients and HCs (238, 239). In one of these studies involving 31 patients with SLE, patients displaying high IFN-α bioactivity (58.1% of them) had a higher frequency of Th17 cells in peripheral blood than those with low IFN-α bioactivity (mean ± SD 1.9 ± 1.0 vs. 1.2 ± 0.9). Additionally, subjects with high IFN-α bioactivity and elevated Th17 cells had significantly higher disease activity and serum IL-6 levels than those with low IFN-α and Th17 cells. Suggesting that IFN-α and Th17 cell pathways co-exist and co-regulate the disease pathogenesis (238). Other studies found a significant correlation between the Th17/IL-17 axis and B-lymphocyte stimulator (BLyS/BAFF), a factor strongly correlated with IFN type I (239, 240). In another study involving 33 patients with cutaneous lupus erythematosus who underwent biopsy, the level of IL-17A in tissue correlated positively with the IFN-α expression (Spearman's ρ = 0.56) (20).

The repercussion of this relationship between Type I IFN and the Th17/IL-17 axis in kidney damage was evident in an experimental model in which mice deficient in IL-17RA were protected from Type I Interferon-dependent crescentic glomerulonephritis. This effect was associated with impaired renal infiltration by activated macrophages, despite unaffected systemic response (147). As the underlying mechanism, the authors have shown that IL-17 in association with IFN-I differentially regulates the expression of macrophage chemoattractants genes, including Ccl2 (encoding CCL2) in RTEC (147). However, no primary study evaluated this relationship in LN in humans. A gap in knowledge to be filled in next studies, and combining genetic studies with integrative Bayesian network approaches may bring additional information to current knowledge in this disease characterized by heterogeneity (209).

The Th17/IL-17 axis may be the bridge (or part of it) between LES and other organs comorbidities and outcomes (241, 242), such as cardiovascular disease because it is known to promote endothelial activation (233, 234), prothrombotic states (167, 168), hypertension (166, 177), and atherogenesis (207, 243, 244); and osteoporosis because it is known to increase bone catabolic activity (245).

## The Potential of Targeting the Th17/IL-17 Axis and Related Pathways on Nephroprotection

As described above, the Th17/IL-17 axis is involved in several points in the renal damage chain. Its effects include the induction of changes in the cytoskeleton of the podocytes with increased motility, decreased expression of the podocyte health proteins, increased oxidative stress, activation of inflammasome and caspases, and induction of podocytes apoptosis. The axis also promotes the activation of the profibrotic pathways, such as increasing the expression of TGF-β and the promotion of EMT with consequent increase of extracellular matrix proteins. In addition, it stimulates the synthesis of inflammatory cytokines by intrinsic and immune cells, synthesis of growth factors and chemokines which together result in granulopoiesis/myelopoiesis, and recruitment of more inflammatory cells. Therefore, inhibition of the Th17/IL-17 axis (and its signaling pathways) represents a promising strategy in treating lupus nephritis in an early view.

### Agents Targeting Directly the Th17/IL-17 Axis

Several agents directly interfere with the axis and are approved to treat diseases in which the Th17/IL-17 axis clearly drives the inflammation. So, secukinumab and ixekizumab are agents targeting IL17A, both approved for ankylosing spondylitis, plaque psoriasis, and psoriatic arthritis (246); Bimekizumab that neutralizes both IL-17A and IL-17F is in preclinical phases for psoriatic arthritis and ankylosing spondylitis (247) and brodalumab an anti-IL17R, is approved for plaque psoriasis (248). Regarding the use of these agents in lupus, there are no studies completed so far; however, there are two ongoing trials to assess the safety, efficacy, and tolerability of secukinumab in patients with active lupus nephritis (NCT04181762); and the safety and efficacy of secukinumab in cutaneous manifestation of lupus (NCT03866317). In a case report involving a patient with lupus nephritis complicated by psoriasis vulgaris, the use of secukinumab was reported to be effective for both conditions with improvement in clinical and laboratory parameters (249).

### Agents Targeting Indirectly the Th17/IL-17 Axis and Related Pathways

The Th17/IL-17 axis can be targeted indirectly in several ways, from interfering in the differentiating pathways, inhibition of migratory capacity, acting on mechanisms that favor polarization, including immunometabolism.

The differentiating pathways of Th17 cells are also a therapeutic target to be explored to prevent the prosperity of the axis. In fact, in a clinical trial, the addition of ustekinumab, a human monoclonal antibody against IL-12 and IL-23, to standard care resulted in better efficacy in clinical and laboratory parameters (250). It is noteworthy that il-23 not only drives the expansion, survival of pathogenic Th17 and other IL-17-producing cells (84) but also decreases Treg by decreasing the production of IL-2 (the positive regulator of Treg) (251). Thus, the beneficial effect of its inhibition should involve as mechanisms the decrease of Th17 and the increase of Treg, the impairement of the IL-23/IL-17 synergisms, among other potential mechanisms. Still, on the path of differentiation, the inhibition of STAT3, the main Signal transducer in Th17 differentiation, delayed/limited the installation of lupus nephritis in experimental models (252–254). In another experimental model of LN, renal pathological damage was attenuated with the use of α-mangostin and 3β-acetyloxy-oleanolic, compounds with inhibitory activity on retinoic acid receptor-related orphan receptor gamma t (RORγt), the transcription factor for Th17 differentiation. These compounds significantly decreased serum anti-dsDNA antibody levels, IL-17A, and IFN-γ expression (255, 256).

Since metabolic changes at the level of T cells are important in Th17 polarization and immune hyperreactivity, targeting the immunometabolism is another potentially promissory indirect strategy in SLE (110, 111). In experimental studies, metformin, which inhibits oxygen consumption and glucose oxidation, inhibited the activation of T cells, with a consequent decrease in the production of IFN-γ and IL-17 (257, 258). In another study with glucose transport inhibitors (CG-5), there was a decrease in Th1 and Th17 polarization by inhibiting their differentiation, accompanied by induction of regulatory T (Treg) (259). In addition to the effect on T cells, CG-5 treatment reduced the expansion of B cells in GC and autoantibodies' production (259). However, in a clinical trial, the addition of metformin to standard care could not demonstrate an additional benefit in reducing SLE recurrence (260). The hyperactivation of the mTOR pathway, a feature that favors Th17 polarization, is another potential target. In two studies with SLE patients, the use of rapamycin (an inhibitor of mTOR pathway) in combination with IL-2 or all-trans retinoic acid (ATRA) showed clinical efficacy decreasing the disease activity, associated with reduced Th17 cells, and restoration and long-term maintenance of Treg/Th17 ratio balance (261, 262). Aligned with this data, in a 12-months prospective open-label study, rapamycin significantly reduced the disease activity scores (SLEDAI and BILAG), associated with a reduction in IL-17 production (either by Th17 cells or double-negative T cells) (263). A trial is registered to assess the efficacy and safety of rapamycin in patients with active SLE (NCT04582136).

Several other agents have shown their potential in improving SLE interfering with the axis. Thus, the immunomodulatory efficacy of stem cell therapies (either Umbilical cord, Bone Marrow or adipose-derived) involves the suppression of the axis or restoration of the Treg/Th17 balance (12, 154); and defects in the functioning of stem cells trigger the disease (121). The beneficial effect of specific MicroRNA as miR-125a-3p and MicroRNA-10a-3p also involve interference on the axis (153, 264). In an experimental study, punicalagin (a bioactive antagonist of PAR2) ameliorated lupus nephritis, in association with a significant reduction in splenic Th17 populations compared to the vehicle controls (265). Remembering that PARs are involved in Th17-induced rearrangement in the cytoskeleton and increased permeability (136). In an experimental study with MRL/lpr mice, a traditional Chinese medicinal formula suppressed the IL-17 production and Th17 activity by inhibiting the expression of CaMK4, which was associated with a decrease in renal hypercellularity and infiltration by neutrophils (266). It is worth remembering that CaMK4 is involved in Th17 activity and enhances its migratory capacity (96).

### The Effect of Lupus Current Medicines on Th17/IL-17 Axis and Related Pathways

Many of the drugs with just known efficacy in the treatment of lupus, and which act on other biological targets, have a parallel effect on the Th17/IL-17 axis. Thus, for example, the effect of methylprednisolone on improving lupus nephritis was also associated with the rebalancing of Splenic CD4+ cells with a significant reduction in Th17 populations compared to controls in an experimental study (265). This corroborates the clinical observation that induction treatment was associated with the progressive reduction of IL-17A, IL-6, and IL-21 (50). Hydroxychloroquine, an immunomodulator in lupus, inhibited Th17 differentiation (267), and reduced Th17-related cytokines in patients (268). Mycophenolic acid, used in an experimental study, inhibited the production of IL-17A, which occurred with the reduction of granulopoiesis; and this effect was completely abolished in mice lacking the IL-17 receptor (269). The same drug showed an effect of reducing STAT3 phosphorylation in patients with SLE (270), which is crucial in synthesizing IL-17 and IL-21 (84). Even Belimumab, a recombinant human IgG-1λ monoclonal antibody that inhibits B-cell activating factor (BLyS/BAFF), effective in lupus nephritis (271), shown to occur, in its effectiveness, with the restoration of the Treg/Th17 balance (272).

## Conclusions and Future Directions

Dysregulated immunity at the Th17/IL-17 axis level plays a significant role in lupus nephritis pathogenesis and ongoing damage, following the initial activation of APC by immunogenic DNA or DNA-containing immune complexes. The Th17/IL-17 axis orchestrates a chain of events that promote a proinflammatory and profibrotic environment stimulating intrinsic renal and resident immune cells to synthesize inflammatory cytokines and chemokines, promoting further recruitment of immune cells into the kidney. The Th17/IL-17 axis also exercises this driver and amplifier role systemically. All resident kidney cells express receptors for IL-17 and respond to IL-17 exposure in many ways, including changes on the cytoskeleton with increased motility, decreased expression of health proteins, increased oxidative stress, and activation of the inflammasome and caspases resulting in podocytes apoptosis. In renal tubular epithelial cells, IL-17 increases the expression of profibrotic and proinflammatory factors, such as TGF-β and fibronectin; and probably induces EMT of RTEC, promoting the further synthesis of the extracellular matrix, with all consequent changes in microstructure and renal functioning.

Despite considerable evidence on the contribution of the Th17/IL-17 axis in the pathogenesis of NL, studies directed to the Th17/IL-17 axis as a therapeutic target did not change the course of the disease as expected- a real gap in translation from bench to bedside. More works are needed to dissect the role of the Th17/IL-17 axis in the pathogenesis of the disease, and the underlying signaling pathways, to open the opportunity to target it effectively, preferably in a multitarget instead of single-cell based approach. In addition, clinical trials with the best designs are necessary, taking into account the clinical and immunological heterogeneity that characterize lupus.

## Author Contributions

FP wrote the manuscript and prepared figures. HA provided critical comments and revised the text. All authors contributed to the article and approved the submitted version.

## Conflict of Interest

The authors declare that the research was conducted in the absence of any commercial or financial relationships that could be construed as a potential conflict of interest.

## Publisher's Note

All claims expressed in this article are solely those of the authors and do not necessarily represent those of their affiliated organizations, or those of the publisher, the editors and the reviewers. Any product that may be evaluated in this article, or claim that may be made by its manufacturer, is not guaranteed or endorsed by the publisher.

## References

[1] Talaat RM Mohamed SF Bassyouni IH Raouf AA. Th1/Th2/Th17/Treg cytokine imbalance in systemic lupus erythematosus (SLE) patients: correlation with disease activity. Cytokine. (2015) 72:146–53. 10.1016/j.cyto.2014.12.027 25647269

[2] Choi S-C Xu Z Li W Yang H Roopenian DC Morse HC 3rd. Relative contributions of B cells and dendritic cells from lupus-prone mice to CD4 T cell polarization. J Immunol. (2018) 200:3087–99. 10.4049/jimmunol.1701179 29563177 PMC5915918

[3] Kono DH Haraldsson MK Lawson BR Pollard KM Koh YT Du X. Endosomal TLR signaling is required for anti-nucleic acid and rheumatoid factor autoantibodies in lupus. Proc Natl Acad Sci USA. (2009) 106:12061–6. 10.1073/pnas.0905441106 19574451 PMC2715524

[4] Lande R Ganguly D Facchinetti V Frasca L Conrad C Gregorio J. Neutrophils activate plasmacytoid dendritic cells by releasing self-DNA-peptide complexes in systemic lupus erythematosus. Sci Transl Med. (2011) 3:73ra19. 10.1126/scitranslmed.3001180 21389263 PMC3399524

[5] Villanueva E Yalavarthi S Berthier CC Hodgin JB Khandpur R Lin AM. Netting neutrophils induce endothelial damage, infiltrate tissues, and expose immunostimulatory molecules in systemic lupus erythematosus. J Immunol. (2011) 187:538–52. 10.4049/jimmunol.1100450 21613614 PMC3119769

[6] Tsokos GC. Autoimmunity and organ damage in systemic lupus erythematosus. Nat Immunol. (2020) 21:605–14. 10.1038/s41590-020-0677-6 32367037 PMC8135909

[7] Shah K Lee W-W Lee S-H Kim SH Kang SW Craft J. Dysregulated balance of Th17 and Th1 cells in systemic lupus erythematosus. Arthritis Res Ther. (2010) 12:R53. 10.1186/ar2964 20334681 PMC2888202

[8] Menon M Blair PA Isenberg DA Mauri C. A regulatory feedback between plasmacytoid dendritic cells and regulatory B cells is aberrant in systemic lupus erythematosus. Immunity. (2016) 44:683–97. 10.1016/j.immuni.2016.02.012 26968426 PMC4803914

[9] Feldman CH Hiraki LT Liu J Fischer MA Solomon DH Alarcón GS. Epidemiology and sociodemographics of systemic lupus erythematosus and lupus nephritis among US adults with Medicaid coverage, 2000-2004. Arthritis Rheum. (2013) 65:753–63. 10.1002/art.37795 23203603 PMC3733212

[10] Lim SS Bayakly AR Helmick CG Gordon C Easley KA Drenkard C. The incidence and prevalence of systemic lupus erythematosus, 2002-2004: The Georgia Lupus Registry. Arthritis Rheumatol. (2014) 66:357–68. 10.1002/art.38239 24504808 PMC4617771

[11] Tsokos GC Lo MS Costa Reis P Sullivan KE. New insights into the immunopathogenesis of systemic lupus erythematosus. Nat Rev Rheumatol. (2016) 12:716–30. 10.1038/nrrheum.2016.186 27872476

[12] Wang D Huang S Yuan X Liang J Xu R Yao G. The regulation of the Treg/Th17 balance by mesenchymal stem cells in human systemic lupus erythematosus. Cell Mol Immunol. (2017) 14:423–31. 10.1038/cmi.2015.89 26435067 PMC5423084

[13] Álvarez-Rodríguez L Martínez-Taboada V Calvo-Alén J Beares I Villa I López-Hoyos M. Altered Th17/Treg ratio in peripheral blood of systemic lupus erythematosus but not primary antiphospholipid syndrome. Front Immunol. (2019) 10:391. 10.3389/fimmu.2019.00391 30894863 PMC6414457

[14] Henriques A Inês L Couto M Pedreiro S Santos C Magalhães M. Frequency and functional activity of Th17, Tc17 and other T-cell subsets in systemic lupus erythematosus. Cell Immunol. (2010) 264:97–103. 10.1016/j.cellimm.2010.05.004 20553755

[15] Rother N van der Vlag J. Disturbed T cell signaling and altered Th17 and regulatory T cell subsets in the pathogenesis of systemic lupus erythematosus. Front Immunol. (2015) 6:610. 10.3389/fimmu.2015.00610 26648939 PMC4663269

[16] Mok MY Wu HJ Lo Y Lau CS. The relation of interleukin 17 (IL-17) and IL-23 to Th1/Th2 cytokines and disease activity in systemic lupus erythematosus. J Rheumatol. (2010) 37:2046–52. 10.3899/jrheum.100293 20682672

[17] Ma J Yu J Tao X Cai L Wang J Zheng SG. The imbalance between regulatory and IL-17-secreting CD4+ T cells in lupus patients. Clin Rheumatol. (2010) 29:1251–8. 10.1007/s10067-010-1510-7 20563617

[18] Ichinose K Arima K Ushigusa T Nishino A Nakashima Y Suzuki T. Distinguishing the cerebrospinal fluid cytokine profile in neuropsychiatric systemic lupus erythematosus from other autoimmune neurological diseases. Clin Immunol. (2015) 157:114–20. 10.1016/j.clim.2015.01.010 25656641

[19] Vincent FB Northcott M Hoi A Mackay F Morand EF. Clinical associations of serum interleukin-17 in systemic lupus erythematosus. Arthritis Res Ther. (2013) 15:R97. 10.1186/ar4277 23968496 PMC3979031

[20] Oh SH Roh HJ Kwon JE Lee SH Kim JY Choi HJ. Expression of interleukin-17 is correlated with interferon-α expression in cutaneous lesions of lupus erythematosus. Clin Exp Dermatol. (2011) 36:512–20. 10.1111/j.1365-2230.2010.03996.x 21631571

[21] Peters JH Tjabringa GS Fasse E de Oliveira VL Schalkwijk J Koenen HJPM. Co-culture of healthy human keratinocytes and T-cells promotes keratinocyte chemokine production and RORγt-positive IL-17 producing T-cell populations. J Dermatol Sci. (2013) 69:44–53. 10.1016/j.jdermsci.2012.10.004 23127421

[22] Tsanaktsi A Solomou EE Liossis S-NC. Th1/17 cells, a subset of Th17 cells, are expanded in patients with active systemic lupus erythematosus. Clin Immunol. (2018) 195:101–6. 10.1016/j.clim.2018.08.005 30118866

[23] Paust H-J Turner J-E Steinmetz OM Peters A Heymann F Hölscher C. The IL-23/Th17 axis contributes to renal injury in experimental glomerulonephritis. J Am Soc Nephrol. (2009) 20:969–79. 10.1681/ASN.2008050556 19339380 PMC2678032

[24] Pisitkun P Ha H-L Wang H Claudio E Tivy CC Zhou H. Interleukin-17 cytokines are critical in development of fatal lupus glomerulonephritis. Immunity. (2012) 37:1104–15. 10.1016/j.immuni.2012.08.014 23123062 PMC3594848

[25] Kitching AR Holdsworth SR. The emergence of TH17 cells as effectors of renal injury. J Am Soc Nephrol. (2011) 22:235–8. 10.1681/ASN.2010050536 21289213

[26] Turner J-E Paust H-J Steinmetz OM Panzer U. The Th17 immune response in renal inflammation. Kidney Int. (2010) 77:1070–5. 10.1038/ki.2010.102 20375986

[27] Lubberts E. The IL-23-IL-17 axis in inflammatory arthritis. Nat Rev Rheumatol. (2015) 11:415–29. 10.1038/nrrheum.2015.53 25907700

[28] Jadidi-Niaragh F Mirshafiey A. Th17 cell, the new player of neuroinflammatory process in multiple sclerosis. Scand J Immunol. (2011) 74:1–13. 10.1111/j.1365-3083.2011.02536.x 21338381

[29] Dolff S Witzke O Wilde B. Th17 cells in renal inflammation and autoimmunity. Autoimmun Rev. (2019) 18:129–36. 10.1016/j.autrev.2018.08.006 30572135

[30] Krebs CF Schmidt T Riedel J-H Panzer U. T helper type 17 cells in immune-mediated glomerular disease. Nat Rev Nephrol. (2017) 13:647–59. 10.1038/nrneph.2017.112 28781371

[31] Hirai Y Iyoda M Shibata T Kuno Y Kawaguchi M Hizawa N. IL-17A stimulates granulocyte colony-stimulating factor production via ERK1/2 but not p38 or JNK in human renal proximal tubular epithelial cells. Am J Physiol Renal Physiol. (2012) 302:F244–50. 10.1152/ajprenal.00113.2011 21993883

[32] Iyoda M Shibata T Kawaguchi M Hizawa N Yamaoka T Kokubu F. IL-17A and IL-17F stimulate chemokines via MAPK pathways (ERK1/2 and p38 but not JNK) in mouse cultured mesangial cells: synergy with TNF-alpha and IL-1beta. Am J Physiol Renal Physiol. (2010) 298:F779–87. 10.1152/ajprenal.00198.2009 20042461

[33] Krohn S Nies JF Kapffer S Schmidt T Riedel J-H Kaffke A. IL-17C/IL-17 receptor E signaling in CD4 T cells promotes T17 cell-driven glomerular inflammation. J Am Soc Nephrol. (2018) 29:1210–22. 10.1681/ASN.2017090949 29483158 PMC5875959

[34] Yuan S Zhang S Zhuang Y Zhang H Bai J Hou Q. Interleukin-17 stimulates STAT3-mediated endothelial cell activation for neutrophil recruitment. Cell Physiol Biochem. (2015) 36:2340–56. 10.1159/000430197 26279438

[35] Ge D You Z. Expression of interleukin-17RC protein in normal human tissues. Int Arch Med. (2008) 1:19. 10.1186/1755-7682-1-19 18928529 PMC2596096

[36] Peng X Xiao Z Zhang J Li Y Dong Y Du J. IL-17A produced by both γδ T and Th17 cells promotes renal fibrosis via RANTES-mediated leukocyte infiltration after renal obstruction. J Pathol. (2015) 235:79–89. 10.1002/path.4430 25158055

[37] Mehrotra P Collett JA McKinney SD Stevens J Ivancic CM Basile DP. IL-17 mediates neutrophil infiltration and renal fibrosis following recovery from ischemia reperfusion: compensatory role of natural killer cells in athymic rats. Am J Physiol Renal Physiol. (2017) 312:F385–97. 10.1152/ajprenal.00462.2016 27852609 PMC5374313

[38] Liu Y Wang K Liang X Li Y Zhang Y Zhang C. Complement C3 produced by macrophages promotes renal fibrosis via IL-17A secretion. Front Immunol. (2018) 9:2385. 10.3389/fimmu.2018.02385 30405606 PMC6204358

[39] Weng C-H Li Y-J Wu H-H Liu S-H Hsu H-H Chen Y-C. Interleukin-17A induces renal fibrosis through the ERK and Smad signaling pathways. Biomed Pharmacother. (2020) 123:109741. 10.1016/j.biopha.2019.109741 31901549

[40] Coto E Gómez J Suárez B Tranche S Díaz-Corte C Ortiz A. Association between the IL17RA rs4819554 polymorphism and reduced renal filtration rate in the Spanish RENASTUR cohort. Hum Immunol. (2015) 76:75–8. 10.1016/j.humimm.2015.01.027 25636567

[41] Jacob N Yang H Pricop L Liu Y Gao X Zheng SG. Accelerated pathological and clinical nephritis in systemic lupus erythematosus-prone New Zealand Mixed 2328 mice doubly deficient in TNF receptor 1 and TNF receptor 2 via a Th17-associated pathway. J Immunol. (2009) 182:2532–41. 10.4049/jimmunol.0802948 19201910 PMC2790862

[42] Zickert A Amoudruz P Sundström Y Rönnelid J Malmström V Gunnarsson I. IL-17 and IL-23 in lupus nephritis - association to histopathology and response to treatment. BMC Immunol. (2015) 16:7. 10.1186/s12865-015-0070-7 25887118 PMC4326189

[43] Chen D-Y Chen Y-M Wen M-C Hsieh T-Y Hung W-T Lan J-L. The potential role of Th17 cells and Th17-related cytokines in the pathogenesis of lupus nephritis. Lupus. (2012) 21:1385–96. 10.1177/0961203312457718 22892208

[44] Xia LP Li BF Shen H Lu J. Interleukin-27 and interleukin-23 in patients with systemic lupus erythematosus: possible role in lupus nephritis. Scand J Rheumatol. (2015) 44:200–5. 10.3109/03009742.2014.962080 25562331

[45] Dedong H Feiyan Z Jie S Xiaowei L Shaoyang W. Analysis of interleukin-17 and interleukin-23 for estimating disease activity and predicting the response to treatment in active lupus nephritis patients. Immunol Lett. (2019) 210:33–9. 10.1016/j.imlet.2019.04.002 31004679

[46] Steinmetz OM Summers SA Gan P-Y Semple T Holdsworth SR Kitching AR. The Th17-defining transcription factor RORγt promotes glomerulonephritis. J Am Soc Nephrol. (2011) 22:472–83. 10.1681/ASN.2010040435 21183590 PMC3060441

[47] Kluger MA Nosko A Ramcke T Goerke B Meyer MC Wegscheid C. RORγt expression in T promotes systemic lupus erythematosus via IL-17 secretion, alteration of T phenotype and suppression of Th2 responses. Clin Exp Immunol. (2017) 188:63–78. 10.1111/cei.12905 27880975 PMC5343349

[48] Cheng Y Yang X Zhang X An Z. Analysis of expression levels of IL-17 and IL-34 and influencing factors for prognosis in patients with lupus nephritis. Exp Ther Med. (2019) 17:2279–83. 10.3892/etm.2019.7168 30783486 PMC6364195

[49] Yazici MU Orhan D Kale G Besbas N Ozen S. Studying IFN-gamma, IL-17 and FOXP3 in pediatric lupus nephritis. Pediatr Nephrol. (2014) 29:853–62. 10.1007/s00467-013-2695-1 24482023

[50] Wang N Gao C Cui S Qin Y Zhang C Yi P. Induction therapy downregulates the expression of Th17/Tfh cytokines in patients with active lupus nephritis. Am J Clin Exp Immunol. (2018) 7:67–75. 30245920 PMC6146154

[51] Wang Y Ito S Chino Y Goto D Matsumoto I Murata H. Laser microdissection-based analysis of cytokine balance in the kidneys of patients with lupus nephritis. Clin Exp Immunol. (2010) 159:1–10. 10.1111/j.1365-2249.2009.04031.x 19807734 PMC2802690

[52] Abdel Galil SM Ezzeldin N El-Boshy ME. The role of serum IL-17 and IL-6 as biomarkers of disease activity and predictors of remission in patients with lupus nephritis. Cytokine. (2015) 76:280–7. 10.1016/j.cyto.2015.05.007 26073684

[53] Saber NZ Maroof SH Soliman DA Fathi MS. Expression of T helper 17 cells and interleukin 17 in lupus nephritis patients. Egypt Rheumatol. (2017) 39:151–7. 10.1016/j.ejr.2017.01.005

[54] Nakhjavani M Abediazar S Ghorbanihaghjo A Esmaeili N Pourlak T Vahed SZ. Serum tumor necrosis factor-like weak inducer of apoptosis (sTWEAK) and IL-17 levels are associated with disease activity in systemic lupus erythematosus patients with and without nephritis. J Renal Injury Prevent. (2019) 8:204–10. 10.15171/jrip.2019.38

[55] Susianti H Iriane VM Dharmanata S Handono K Widijanti A Gunawan A. Analysis of urinary TGF-β1, MCP-1, NGAL, and IL-17 as biomarkers for lupus nephritis. Pathophysiology. (2015) 22:65–71. 10.1016/j.pathophys.2014.12.003 25595582

[56] Sigdel KR Duan L Wang Y Hu W Wang N Sun Q. Serum cytokines Th1, Th2, and Th17 expression profiling in active lupus nephritis-IV: from a Southern Chinese Han population. Mediators Inflamm. (2016) 2016:4927530. 10.1155/2016/4927530 27738386 PMC5055982

[57] Xing Q Wang B Su H Cui J Li J. Elevated Th17 cells are accompanied by FoxP3+ Treg cells decrease in patients with lupus nephritis. Rheumatol Int. (2012) 32:949–958. 10.1007/s00296-010-1771-0 21243492

[58] Dong G Ye R Shi W Liu S Wang T Yang X. IL-17 induces autoantibody overproduction and peripheral blood mononuclear cell overexpression of IL-6 in lupus nephritis patients. Chin Med J. (2003) 116:543–8. 12875719

[59] Cavalcanti A Santos R Mesquita Z Duarte ALBP Lucena-Silva N. Cytokine profile in childhood-onset systemic lupus erythematosus: a cross-sectional and longitudinal study. Braz J Med Biol Res. (2017) 50:e5738. 10.1590/1414-431x20175738 28380214 PMC5423750

[60] Kshirsagar S Riedl M Billing H Tönshoff B Thangavadivel S Steuber C. Akt-dependent enhanced migratory capacity of Th17 cells from children with lupus nephritis. J Immunol. (2014) 193:4895–903. 10.4049/jimmunol.1400044 25339666

[61] Peliçari K de O Postal M Sinicato NA Peres FA Fernandes PT Marini R. Serum interleukin-17 levels are associated with nephritis in childhood-onset systemic lupus erythematosus. Clinics. (2015) 70:313–7. 10.6061/clinics/2015(05)01 26039945 PMC4449477

[62] AlFadhli S AlFailakawi A 'a Ghanem AAM. Th-17 related regulatory network in the pathogenesis of Arab patients with systemic lupus erythematosus and lupus nephritis. Int J Rheum Dis. (2016) 19:512–20. 10.1111/1756-185X.12393 25496163

[63] Jakiela B Kosałka J Plutecka H Bazan-Socha S Sanak M Musiał J. Facilitated expansion of Th17 cells in lupus nephritis patients. Clin Exp Immunol. (2018) 194:283–94. 10.1111/cei.13196 30086206 PMC6230997

[64] Edelbauer M Kshirsagar S Riedl M Billing H Tönshoff B Haffner D. Activity of childhood lupus nephritis is linked to altered T cell and cytokine homeostasis. J Clin Immunol. (2012) 32:477–87. 10.1007/s10875-011-9637-0 22228566

[65] Elkoumi MA Allah MA Mohamed FY Boraey NF Abdellatif SH Shehab MM. Association of interleukin-17A gene polymorphisms and susceptibility to systemic lupus erythematosus in Egyptian children and adolescents: a multi-centre study. Lupus. (2020) 29:767–75. 10.1177/0961203320922305 32380889

[66] Rastin M Soltani S Nazemian F Sahebari M Mirfeizi SZ Tabasi N. Expression of T helper 17 and regulatory T cell cytokines and molecules in glomerulonephritis class IV systemic lupus erythematosus. Iran J Kidney Dis. (2016) 10:113–8. 27225718

[67] Soni C Perez OA Voss WN Pucella JN Serpas L Mehl J. Plasmacytoid dendritic cells and type i interferon promote extrafollicular B cell responses to extracellular self-DNA. Immunity. (2020) 52:1022–38.e7. 10.1016/j.immuni.2020.04.015 32454024 PMC7306002

[68] Wang H Li T Chen S Gu Y Ye S. Neutrophil extracellular trap mitochondrial DNA and its autoantibody in systemic lupus erythematosus and a proof-of-concept trial of metformin. Arthritis Rheumatol. (2015) 67:3190–200. 10.1002/art.39296 26245802

[69] Summers SA Hoi A Steinmetz OM O'Sullivan KM Ooi JD Odobasic D. TLR9 and TLR4 are required for the development of autoimmunity and lupus nephritis in pristane nephropathy. J Autoimmun. (2010) 35:291–8. 10.1016/j.jaut.2010.05.004 20810248

[70] Pérez-Ferro M Serrano Del Castillo C Sánchez-Pernaute O. Cell membrane-bound TLR2 and TLR4: potential predictors of active systemic lupus erythematosus and lupus nephritis. J Rheumatol. (2016) 43:1444–5. 10.3899/jrheum.151386 27371649

[71] Ma K Li J Wang X Lin X Du W Yang X. TLR4CXCR4 plasma cells drive nephritis development in systemic lupus erythematosus. Ann Rheum Dis. (2018) 77:1498–506. 10.1136/annrheumdis-2018-213615 29925508

[72] Allam R Scherbaum CR Darisipudi MN Mulay SR Hägele H Lichtnekert J. Histones from dying renal cells aggravate kidney injury via TLR2 and TLR4. J Am Soc Nephrol. (2012) 23:1375–88. 10.1681/ASN.2011111077 22677551 PMC3402284

[73] Herlands RA Christensen SR Sweet RA Hershberg U Shlomchik MJ. T cell-independent and toll-like receptor-dependent antigen-driven activation of autoreactive B cells. Immunity. (2008) 29:249–60. 10.1016/j.immuni.2008.06.009 18691914 PMC4106705

[74] Pawar RD Ramanjaneyulu A Kulkarni OP Lech M Segerer S Anders H-J. Inhibition of Toll-like receptor-7 (TLR-7) or TLR-7 plus TLR-9 attenuates glomerulonephritis and lung injury in experimental lupus. J Am Soc Nephrol. (2007) 18:1721–31. 10.1681/ASN.2006101162 17460144

[75] Sakata K Nakayamada S Miyazaki Y Kubo S Ishii A Nakano K. Up-regulation of TLR7-mediated IFN-α production by plasmacytoid dendritic cells in patients with systemic lupus erythematosus. Front Immunol. (2018) 9:1957. 10.3389/fimmu.2018.01957 30210502 PMC6121190

[76] Dieker J Tel J Pieterse E Thielen A Rother N Bakker M. Circulating apoptotic microparticles in systemic lupus erythematosus patients drive the activation of dendritic cell subsets and prime neutrophils for NETosis. Arthritis Rheumatol. (2016) 68:462–472. 10.1002/art.39417 26360137

[77] Hammer GE Ma A. Molecular control of steady-state dendritic cell maturation and immune homeostasis. Annu Rev Immunol. (2013) 31:743–91. 10.1146/annurev-immunol-020711-074929 23330953 PMC4091962

[78] Segura E Touzot M Bohineust A Cappuccio A Chiocchia G Hosmalin A. Human inflammatory dendritic cells induce Th17 cell differentiation. Immunity. (2013) 38:336–48. 10.1016/j.immuni.2012.10.018 23352235

[79] Brucklacher-Waldert V Ferreira C Stebegg M Fesneau O Innocentin S Marie JC. Cellular stress in the context of an inflammatory environment supports TGF-β-independent T helper-17 differentiation. Cell Rep. (2017) 19:2357–70. 10.1016/j.celrep.2017.05.052 28614720 PMC5483510

[80] Zhou L Ivanov II Spolski R Min R Shenderov K Egawa T. IL-6 programs T(H)-17 cell differentiation by promoting sequential engagement of the IL-21 and IL-23 pathways. Nat Immunol. (2007) 8:967–74. 10.1038/ni1488 17581537

[81] Yang XO Pappu BP Nurieva R Akimzhanov A Kang HS Chung Y. T helper 17 lineage differentiation is programmed by orphan nuclear receptors ROR alpha and ROR gamma. Immunity. (2008) 28:29–39. 10.1016/j.immuni.2007.11.016 18164222 PMC2587175

[82] Podojil JR Miller SD. Molecular mechanisms of T-cell receptor and costimulatory molecule ligation/blockade in autoimmune disease therapy. Immunol Rev. (2009) 229:337–55. 10.1111/j.1600-065X.2009.00773.x 19426232 PMC2845642

[83] Pacheco GV Novelo Noh IB Velasco Cárdenas RM-H Angulo Ramírez AV López Villanueva RF Quintal Ortiz IG. Expression of TLR-7, MyD88, NF-kB, and INF-α in B lymphocytes of Mayan women with systemic lupus erythematosus in Mexico. Front Immunol. (2016) 7:22. 10.3389/fimmu.2016.00022 26870038 PMC4735402

[84] Lee S Nakayamada S Kubo S Yamagata K Yoshinari H Tanaka Y. Interleukin-23 drives expansion of Thelper 17 cells through epigenetic regulation by signal transducer and activators of transcription 3 in lupus patients. Rheumatology. (2020) 59:3058–3069. 10.1093/rheumatology/keaa176 32375179

[85] Chen S-Y Liu M-F Kuo P-Y Wang C-R. Upregulated expression of STAT3/IL-17 in patients with systemic lupus erythematosus. Clin Rheumatol. (2019) 38:1361–6. 10.1007/s10067-019-04467-8 30767092

[86] Riedel J-H Paust H-J Krohn S Turner J-E Kluger MA Steinmetz OM. IL-17F promotes tissue injury in autoimmune kidney diseases. J Am Soc Nephrol. (2016) 27:3666–77. 10.1681/ASN.2015101077 27030744 PMC5118482

[87] Wang F Yin J Lin Y Zhang F Liu X Zhang G. IL-17C has a pathogenic role in kidney ischemia/reperfusion injury. Kidney Int. (2020) 97:1219–29. 10.1016/j.kint.2020.01.015 32331702

[88] Cortvrindt C Speeckaert R Moerman A Delanghe JR Speeckaert MM. The role of interleukin-17A in the pathogenesis of kidney diseases. Pathology. (2017) 49:247–58. 10.1016/j.pathol.2017.01.003 28291548

[89] Yu H Cui S Mei Y Li Q Wu L Duan S. Mesangial cells exhibit features of antigen-presenting cells and activate CD4+ T cell responses. J Immunol Res. (2019) 2019:2121849. 10.1155/2019/2121849 31317046 PMC6604415

[90] Machida H Ito S Hirose T Takeshita F Oshiro H Nakamura T. Expression of Toll-like receptor 9 in renal podocytes in childhood-onset active and inactive lupus nephritis. Nephrol Dial Transplant. (2010) 25:2530–537. 10.1093/ndt/gfq058 20181802

[91] Wu H Chen G Wyburn KR Yin J Bertolino P Eris JM. TLR4 activation mediates kidney ischemia/reperfusion injury. J Clin Invest. (2007) 117:2847–59. 10.1172/JCI31008 17853945 PMC1974864

[92] Xia H Bao W Shi S. Innate immune activity in glomerular podocytes. Front Immunol. (2017) 8:122. 10.3389/fimmu.2017.00122 28228761 PMC5296344

[93] Li S Liu Y He Y Rong W Zhang M Li L. Podocytes present antigen to activate specific T cell immune responses in inflammatory renal disease. J Pathol. (2020) 252:165–77. 10.1002/path.5508 32686090

[94] Yuan D-H Jia Y Hassan OM Xu L-Y Wu X-C. LPS-treated podocytes polarize naive CD4 T cells into Th17 and Treg cells. Biomed Res Int. (2020) 2020:8587923. 10.1155/2020/8587923 32509873 PMC7251438

[95] Pindjakova J Hanley SA Duffy MM Sutton CE Weidhofer GA Miller MN. Interleukin-1 accounts for intrarenal Th17 cell activation during ureteral obstruction. Kidney Int. (2012) 81:379–390. 10.1038/ki.2011.348 21975862 PMC3913378

[96] Koga T Otomo K Mizui M Yoshida N Umeda M Ichinose K. Calcium/calmodulin-dependent kinase IV facilitates the recruitment of interleukin-17-producing cells to target organs through the CCR6/CCL20 axis in Th17 cell-driven inflammatory diseases. Arthritis Rheumatol. (2016) 68:1981–8. 10.1002/art.39665 26945541 PMC4963275

[97] Koga T Ichinose K Mizui M Crispín JC Tsokos GC. Calcium/calmodulin-dependent protein kinase IV suppresses IL-2 production and regulatory T cell activity in lupus. J Immunol. (2012) 189:3490–6. 10.4049/jimmunol.1201785 22942433 PMC3448834

[98] Krebs CF Reimers D Zhao Y Paust H-J Bartsch P Nuñez S. Pathogen-induced tissue-resident memory T17 (T17) cells amplify autoimmune kidney disease. Sci Immunol. (2020) 5:eaba4163. 10.1126/sciimmunol.aba4163 32769171

[99] Jenks SA Cashman KS Zumaquero E Marigorta UM Patel AV Wang X. Distinct effector B cells induced by unregulated toll-like receptor 7 contribute to pathogenic responses in systemic lupus erythematosus. Immunity. (2018) 49:725–39.e6. 10.1016/j.immuni.2018.08.015 30314758 PMC6217820

[100] Zhou H Li B Li J Wu T Jin X Yuan R. Dysregulated T cell activation and aberrant cytokine expression profile in systemic lupus erythematosus. Mediators Inflamm. (2019) 2019:8450947. 10.1155/2019/8450947 31007604 PMC6441516

[101] Yang XO Nurieva R Martinez GJ Kang HS Chung Y Pappu BP. Molecular antagonism and plasticity of regulatory and inflammatory T cell programs. Immunity. (2008) 29:44–56. 10.1016/j.immuni.2008.05.007 18585065 PMC2630532

[102] Gagliani N Amezcua Vesely MC Iseppon A Brockmann L Xu H Palm NW. Th17 cells transdifferentiate into regulatory T cells during resolution of inflammation. Nature. (2015) 523:221–5. 10.1038/nature14452 25924064 PMC4498984

[103] Noack M Miossec P. Th17 and regulatory T cell balance in autoimmune and inflammatory diseases. Autoimmun Rev. (2014) 13:668–77. 10.1016/j.autrev.2013.12.004 24418308

[104] Jiang C Wang H Xue M Lin L Wang J Cai G. Reprograming of peripheral Foxp3 regulatory T cell towards Th17-like cell in patients with active systemic lupus erythematosus. Clin Immunol. (2019) 209:108267. 10.1016/j.clim.2019.108267 31639448

[105] Cui HD Qi ZM Yang LL Qi L Zhang N Zhang XL. Interleukin-10 receptor expression and signalling were down-regulated in CD4^+^ T cells of lupus nephritis patients. Clin Exp Immunol. (2011) 165:163–71. 10.1111/j.1365-2249.2011.04424.x 21635228 PMC3142641

[106] Carter NA Vasconcellos R Rosser EC Tulone C Muñoz-Suano A Kamanaka M. Mice lacking endogenous IL-10-producing regulatory B cells develop exacerbated disease and present with an increased frequency of Th1/Th17 but a decrease in regulatory T cells. J Immunol. (2011) 186:5569–79. 10.4049/jimmunol.1100284 21464089

[107] Zhu H Hu F Sun X Zhang X Zhu L Liu X. CD16 monocyte subset was enriched and functionally exacerbated in driving T-cell activation and B-cell response in systemic lupus erythematosus. Front Immunol. (2016) 7:512. 10.3389/fimmu.2016.00512 27917174 PMC5116853

[108] Crispín JC Vargas-Rojas MI Monsiváis-Urenda A Alcocer-Varela J. Phenotype and function of dendritic cells of patients with systemic lupus erythematosus. Clin Immunol. (2012) 143:45–50. 10.1016/j.clim.2011.12.004 22239954

[109] Sule S Rosen A Petri M Akhter E Andrade F. Abnormal production of pro- and anti-inflammatory cytokines by lupus monocytes in response to apoptotic cells. PLoS ONE. (2011) 6:e17495. 10.1371/journal.pone.0017495 21423726 PMC3056659

[110] Shan J Jin H Xu Y. T cell metabolism: a new perspective on Th17/Treg cell imbalance in systemic lupus erythematosus. Front Immunol. (2020) 11:1027. 10.3389/fimmu.2020.01027 32528480 PMC7257669

[111] Sharabi A Tsokos GC. T cell metabolism: new insights in systemic lupus erythematosus pathogenesis and therapy. Nat Rev Rheumatol. (2020) 16:100–12. 10.1038/s41584-019-0356-x 31949287

[112] Moulton VR Tsokos GC. Abnormalities of T cell signaling in systemic lupus erythematosus. Arthritis Res Ther. (2011) 13:207. 10.1186/ar3251 21457530 PMC3132009

[113] Katsuyama T Tsokos GC Moulton VR. Aberrant T cell signaling and subsets in systemic lupus erythematosus. Front Immunol. (2018) 9:1088. 10.3389/fimmu.2018.01088 29868033 PMC5967272

[114] Coit P Dozmorov MG Merrill JT McCune WJ Maksimowicz-McKinnon K Wren JD. Epigenetic reprogramming in naive CD4+ T cells favoring T cell activation and non-Th1 effector T cell immune response as an early event in lupus flares. Arthritis Rheumatol. (2016) 68:2200–9. 10.1002/art.39720 27111767 PMC5001909

[115] Coit P Jeffries M Altorok N Dozmorov MG Koelsch KA Wren JD. Genome-wide DNA methylation study suggests epigenetic accessibility and transcriptional poising of interferon-regulated genes in naïve CD4+ T cells from lupus patients. J Autoimmun. (2013) 43:78–84. 10.1016/j.jaut.2013.04.003 23623029 PMC3790645

[116] Zhang H Liao X Sparks JB Luo XM. Dynamics of gut microbiota in autoimmune lupus. Appl Environ Microbiol. (2014) 80:7551–60. 10.1128/AEM.02676-14 25261516 PMC4249226

[117] Azzouz D Omarbekova A Heguy A Schwudke D Gisch N Rovin BH. Lupus nephritis is linked to disease-activity associated expansions and immunity to a gut commensal. Ann Rheum Dis. (2019) 78:947–56. 10.1136/annrheumdis-2018-214856 30782585 PMC6585303

[118] López P de Paz B Rodríguez-Carrio J Hevia A Sánchez B Margolles A. Th17 responses and natural IgM antibodies are related to gut microbiota composition in systemic lupus erythematosus patients. Sci Rep. (2016) 6:24072. 10.1038/srep24072 27044888 PMC4820712

[119] Krebs CF Paust H-J Krohn S Koyro T Brix SR Riedel J-H. Autoimmune renal disease is exacerbated by S1P-receptor-1-dependent intestinal Th17 cell migration to the kidney. Immunity. (2016) 45:1078–92. 10.1016/j.immuni.2016.10.020 27851911 PMC6381450

[120] Pan Q Gong L Xiao H Feng Y Li L Deng Z. Basophil activation-dependent autoantibody and interleukin-17 production exacerbate systemic lupus erythematosus. Front Immunol. (2017) 8:348. 10.3389/fimmu.2017.00348 28396669 PMC5366357

[121] Geng L Tang X Wang S Sun Y Wang D Tsao BP. Reduced Let-7f in bone marrow-derived mesenchymal stem cells triggers Treg/Th17 imbalance in patients with systemic lupus erythematosus. Front Immunol. (2020) 11:233. 10.3389/fimmu.2020.00233 32133007 PMC7040072

[122] Panzer U Steinmetz OM Paust H-J Meyer-Schwesinger C Peters A Turner J-E. Chemokine receptor CXCR3 mediates T cell recruitment and tissue injury in nephrotoxic nephritis in mice. J Am Soc Nephrol. (2007) 18:2071–84. 10.1681/ASN.2006111237 17538187

[123] Lu G Zhang X Shen L Qiao Q Li Y Sun J. CCL20 secreted from IgA1-stimulated human mesangial cells recruits inflammatory Th17 cells in IgA nephropathy. PLoS One. (2017) 12:e0178352. 10.1371/journal.pone.0178352 28552941 PMC5446182

[124] Singh SP Zhang HH Foley JF Hedrick MN Farber JM. Human T cells that are able to produce IL-17 express the chemokine receptor CCR6. J Immunol. (2008) 180:214–21. 10.4049/jimmunol.180.1.214 18097022

[125] Steinmetz OM Turner J-E Paust H-J Lindner M Peters A Heiss K. CXCR3 mediates renal Th1 and Th17 immune response in murine lupus nephritis. J Immunol. (2009) 183:4693–704. 10.4049/jimmunol.0802626 19734217

[126] Turner J-E Paust H-J Steinmetz OM Peters A Riedel J-H Erhardt A. CCR6 recruits regulatory T cells and Th17 cells to the kidney in glomerulonephritis. J Am Soc Nephrol. (2010) 21:974–85. 10.1681/ASN.2009070741 20299360 PMC2900961

[127] Ferretti AP Bhargava R Dahan S Tsokos MG Tsokos GC. Calcium/calmodulin kinase IV controls the function of both T cells and kidney resident cells. Front Immunol. (2018) 9:2113. 10.3389/fimmu.2018.02113 30333818 PMC6176098

[128] Petrovic-Djergovic D Popovic M Chittiprol S Cortado H Ransom RF Partida-Sánchez S. CXCL10 induces the recruitment of monocyte-derived macrophages into kidney, which aggravate puromycin aminonucleoside nephrosis. Clin Exp Immunol. (2015) 180:305–15. 10.1111/cei.12579 25561167 PMC4408165

[129] Kanapathippillai P Hedberg A Fenton CG Fenton KA. Nucleosomes contribute to increase mesangial cell chemokine expression during the development of lupus nephritis. Cytokine. (2013) 62:244–52. 10.1016/j.cyto.2013.03.016 23561928

[130] Nastase MV Zeng-Brouwers J Beckmann J Tredup C Christen U Radeke HH. Biglycan, a novel trigger of Th1 and Th17 cell recruitment into the kidney. Matrix Biol. (2018) 68–69:293–317. 10.1016/j.matbio.2017.12.002 29253517

[131] Teichmann LL Cullen JL Kashgarian M Dong C Craft J Shlomchik MJ. Local triggering of the ICOS coreceptor by CD11c(+) myeloid cells drives organ inflammation in lupus. Immunity. (2015) 42:552–65. 10.1016/j.immuni.2015.02.015 25786178 PMC4456685

[132] Odegard JM DiPlacido LD Greenwald L Kashgarian M Kono DH Dong C. ICOS controls effector function but not trafficking receptor expression of kidney-infiltrating effector T cells in murine lupus. J Immunol. (2009) 182:4076–84. 10.4049/jimmunol.0800758 19299705 PMC2746004

[133] Shi H Ye J Teng J Yin Y Hu Q Wu X. Elevated serum autoantibodies against co-inhibitory PD-1 facilitate T cell proliferation and correlate with disease activity in new-onset systemic lupus erythematosus patients. Arthritis Res Ther. (2017) 19:52. 10.1186/s13075-017-1258-4 28274252 PMC5343377

[134] Veldhoen M. Interleukin 17 is a chief orchestrator of immunity. Nat Immunol. (2017) 18:612–621. 10.1038/ni.3742 28518156

[135] Bettelli E Oukka M Kuchroo VK. T(H)-17 cells in the circle of immunity and autoimmunity. Nat Immunol. (2007) 8:345–50. 10.1038/ni0407-345 17375096

[136] May CJ Welsh GI Chesor M Lait PJ Schewitz-Bowers LP Lee RWJ. Human Th17 cells produce a soluble mediator that increases podocyte motility via signaling pathways that mimic PAR-1 activation. Am J Physiol Renal Physiol. (2019) 317:F913–21. 10.1152/ajprenal.00093.2019 31339775 PMC6843047

[137] Liu Y Su L Lin Q Han Y You P Fan Q. Induction of C-Mip by IL-17 plays an important role in adriamycin-induced podocyte damage. Cell Physiol Biochem. (2015) 36:1274–90. 10.1159/000430296 26160339

[138] Moktefi A Zhang S-Y Vachin P Ory V Henique C Audard V. Repression of CMIP transcription by WT1 is relevant to podocyte health. Kidney Int. (2016) 90:1298–311. 10.1016/j.kint.2016.07.016 27650733

[139] Yu L Ye J Liu Q Feng J Gu X Sun Q. c-Maf inducing protein inhibits cofilin-1 activity and alters podocyte cytoskeleton organization. Mol Med Rep. (2017) 16:4955–63. 10.3892/mmr.2017.7156 28791377

[140] Bouachi K Moktefi A Zhang S-Y Oniszczuk J Sendeyo K Remy P. Expression of CMIP in podocytes is restricted to specific classes of lupus nephritis. PLoS ONE. (2018) 13:e0207066. 10.1371/journal.pone.0207066 30439969 PMC6237342

[141] Yan J Li Y Yang H Zhang L Yang B Wang M. Interleukin-17A participates in podocyte injury by inducing IL-1β secretion through ROS-NLRP3 inflammasome-caspase-1 pathway. Scand J Immunol. (2018) 87:e12645. 10.1111/sji.12645 29446486

[142] Zhai S Sun B Zhang Y Zhao L Zhang L. IL-17 aggravates renal injury by promoting podocyte injury in children with primary nephrotic syndrome. Exp Ther Med. (2020) 20:409–17. 10.3892/etm.2020.8698 32537005 PMC7282090

[143] Wang L Li Q Wang L Li C Yang H Wang X. The role of Th17/IL-17 in the pathogenesis of primary nephrotic syndrome in children. Kidney Blood Press Res. (2013) 37:332–45. 10.1159/000350161 24247026

[144] Lavoz C Matus YS Orejudo M Carpio JD Droguett A Egido J. Interleukin-17A blockade reduces albuminuria and kidney injury in an accelerated model of diabetic nephropathy. Kidney Int. (2019) 95:1418–32. 10.1016/j.kint.2018.12.031 30982673

[145] Lee H Lee JW Yoo KD Yoo J-Y Lee JP Kim DK. Cln 3-requiring 9 is a negative regulator of Th17 pathway-driven inflammation in anti-glomerular basement membrane glomerulonephritis. Am J Physiol Renal Physiol. (2016) 311:F505–19. 10.1152/ajprenal.00533.2015 27306982

[146] Zhang Q Luan H Wang L He F Zhou H Xu X. Galectin-9 ameliorates anti-GBM glomerulonephritis by inhibiting Th1 and Th17 immune responses in mice. Am J Physiol Renal Physiol. (2014) 306:F822–32. 10.1152/ajprenal.00294.2013 24477688

[147] Ramani K Biswas PS. Interleukin 17 signaling drives Type I Interferon induced proliferative crescentic glomerulonephritis in lupus-prone mice. Clin Immunol. (2016) 162:31–6. 10.1016/j.clim.2015.10.009 26556529

[148] Ramani K Pawaria S Maers K Huppler AR Gaffen SL Biswas PS. An essential role of interleukin-17 receptor signaling in the development of autoimmune glomerulonephritis. J Leukoc Biol. (2014) 96:463–72. 10.1189/jlb.3A0414-184R 24935958 PMC4138200

[149] Kassianos AJ Wang X Sampangi S Muczynski K Healy H Wilkinson R. Increased tubulointerstitial recruitment of human CD141(hi) CLEC9A(+) and CD1c(+) myeloid dendritic cell subsets in renal fibrosis and chronic kidney disease. Am J Physiol Renal Physiol. (2013) 305:F1391–401. 10.1152/ajprenal.00318.2013 24049150

[150] Disteldorf EM Krebs CF Paust H-J Turner J-E Nouailles G Tittel A. CXCL5 drives neutrophil recruitment in TH17-mediated GN. J Am Soc Nephrol. (2015) 26:55–66. 10.1681/ASN.2013101061 24904089 PMC4279732

[151] Coppock GM Aronson LR Park J Qiu C Park J DeLong JH. Loss of IL-27Rα results in enhanced tubulointerstitial fibrosis associated with elevated Th17 responses. J Immunol. (2020) 205:377–86. 10.4049/jimmunol.1901463 32522836 PMC7368461

[152] Saleh MA Norlander AE Madhur MS. Inhibition of interleukin 17-A but not interleukin-17F signaling lowers blood pressure and reduces end-organ inflammation in angiotensin II-induced hypertension. JACC Basic Transl Sci. (2016) 1:606–16. 10.1016/j.jacbts.2016.07.009 28280792 PMC5337944

[153] Zhang Y Chen X Deng Y. miR-125a-3p decreases levels of interlukin-17 and suppresses renal fibrosis via down-regulating TGF-β1 in systemic lupus erythematosus mediated Lupus nephritic mice. Am J Transl Res. (2019) 11:1843–53. 30972208 PMC6456516

[154] He X Zhang Y Zhu A Zeng K Zhang X Gong L. Suppression of interleukin 17 contributes to the immunomodulatory effects of adipose-derived stem cells in a murine model of systemic lupus erythematosus. Immunol Res. (2016) 64:1157–67. 10.1007/s12026-016-8866-y 27617336

[155] Wenbin Z Guojun G. Resveratrol ameliorates diabetes-induced renal damage through regulating the expression of TGF-β1, collagen IV and Th17/Treg-related cytokines in rats. West Indian Med J. (2014) 63:20–5. 10.7727/wimj.2014.008 25303188 PMC4655635

[156] Wu W-P Tsai Y-G Lin T-Y Wu M-J Lin C-Y. The attenuation of renal fibrosis by histone deacetylase inhibitors is associated with the plasticity of FOXP3IL-17 T cells. BMC Nephrol. (2017) 18:225. 10.1186/s12882-017-0630-6 28693431 PMC5504832

[157] Liu L Li F-G Yang M Wang L Chen Y Wang L. Effect of pro-inflammatory interleukin-17A on epithelial cell phenotype inversion in HK-2 cells*in vitro*. Eur Cytokine Netw. (2016) 27:27–33. 10.1684/ecn.2016.0373 27478076

[158] Dudas PL Sague SL Elloso MM Farrell FX. Proinflammatory/profibrotic effects of interleukin-17A on human proximal tubule epithelium. Nephron Exp Nephrol. (2011) 117:e114–23. 10.1159/000320177 20924205

[159] Wang T Liu Y Zou J-F Cheng Z-S. Interleukin-17 induces human alveolar epithelial to mesenchymal cell transition via the TGF-β1 mediated Smad2/3 and ERK1/2 activation. PLoS ONE. (2017) 12:e0183972. 10.1371/journal.pone.0183972 28873461 PMC5584923

[160] Zhang J Wang D Wang L Wang S Roden AC Zhao H. Profibrotic effect of IL-17A and elevated IL-17RA in idiopathic pulmonary fibrosis and rheumatoid arthritis-associated lung disease support a direct role for IL-17A/IL-17RA in human fibrotic interstitial lung disease. Am J Physiol Lung Cell Mol Physiol. (2019) 316:L487–97. 10.1152/ajplung.00301.2018 30604628

[161] Sisto M Lorusso L Tamma R Ingravallo G Ribatti D Lisi S. Interleukin-17 and−22 synergy linking inflammation and EMT-dependent fibrosis in Sjögren's syndrome. Clin Exp Immunol. (2019) 198:261–72. 10.1111/cei.13337 31165469 PMC6797899

[162] Sisto M Lorusso L Ingravallo G Ribatti D Lisi S. TGFβ1-Smad canonical and -Erk noncanonical pathways participate in interleukin-17-induced epithelial-mesenchymal transition in Sjögren's syndrome. Lab Invest. (2020) 100:824–36. 10.1038/s41374-020-0373-z 31925325

[163] Huang Q Chu S Yin X Yu X Kang C Li X. Interleukin-17A-induced epithelial-mesenchymal transition of human intrahepatic biliary epithelial cells: implications for primary biliary cirrhosis. Tohoku J Exp Med. (2016) 240:269–75. 10.1620/tjem.240.269 27916760

[164] Zepeda-Morales ASM Del Toro-Arreola S García-Benavides L Bastidas-Ramírez BE Fafutis-Morris M Pereira-Suárez AL. Liver fibrosis in bile duct-ligated rats correlates with increased hepatic IL-17 and TGF-β2 expression. Ann Hepatol. (2016) 15:418–26. 10.5604/16652681.1198820 27049496

[165] Liu Q Zhang Y Mao H Chen W Luo N Zhou Q. A crosstalk between the Smad and JNK signaling in the TGF-β-induced epithelial-mesenchymal transition in rat peritoneal mesothelial cells. PLoS ONE. (2012) 7:e32009. 10.1371/journal.pone.0032009 22384127 PMC3288060

[166] Nguyen H Chiasson VL Chatterjee P Kopriva SE Young KJ Mitchell BM. Interleukin-17 causes Rho-kinase-mediated endothelial dysfunction and hypertension. Cardiovasc Res. (2013) 97:696–704. 10.1093/cvr/cvs422 23263331 PMC3583258

[167] Hot A Lenief V Miossec P. Combination of IL-17 and TNFα induces a pro-inflammatory, pro-coagulant and pro-thrombotic phenotype in human endothelial cells. Ann Rheum Dis. (2012) 71:768–76. 10.1136/annrheumdis-2011-200468 22258491

[168] Maione F Parisi A Caiazzo E Morello S D'Acquisto F Mascolo N. Interleukin-17A exacerbates ferric chloride-induced arterial thrombosis in rat carotid artery. Int J Inflam. (2014) 2014:247503. 10.1155/2014/247503 24940514 PMC3997091

[169] Li Y Golden JB Camhi MI Zhang X Fritz Y Diaconu D. Protection from psoriasis-related thrombosis after inhibition of IL-23 or IL-17A. J Invest Dermatol. (2018) 138:310–5. 10.1016/j.jid.2017.09.021 28951241 PMC6693345

[170] Schüler R Brand A Klebow S Wild J Veras FP Ullmann E. Antagonization of IL-17A attenuates skin inflammation and vascular dysfunction in mouse models of psoriasis. J Invest Dermatol. (2019) 139:638–47. 10.1016/j.jid.2018.09.021 30367871

[171] Popovic-Kuzmanovic D Novakovic I Stojanovich L Aksentijevich I Zogovic N Tovilovic G. Increased activity of interleukin-23/interleukin-17 cytokine axis in primary antiphospholipid syndrome. Immunobiology. (2013) 218:186–91. 10.1016/j.imbio.2012.03.002 22559912

[172] Xiao J Zhu F Liu X Xiong J. Th1/Th2/Th17/Treg expression in cultured PBMCs with antiphospholipid antibodies. Mol Med Rep. (2012) 6:1035–9. 10.3892/mmr.2012.1055 22941119

[173] Shaharir SS Mustafar R Mohd R Mohd Said MS Gafor HA. Persistent hypertension in lupus nephritis and the associated risk factors. Clin Rheumatol. (2015) 34:93–7. 10.1007/s10067-014-2802-0 25373448

[174] Ayodele OE Okpechi IG Swanepoel CR. Predictors of poor renal outcome in patients with biopsy-proven lupus nephritis. Nephrology. (2010) 15:482–90. 10.1111/j.1440-1797.2010.01290.x 20609103

[175] Orejudo M Rodrigues-Diez RR Rodrigues-Diez R Garcia-Redondo A Santos-Sánchez L Rández-Garbayo J. Interleukin 17A participates in renal inflammation associated to experimental and human hypertension. Front Pharmacol. (2019) 10:1015. 10.3389/fphar.2019.01015 31572188 PMC6753390

[176] Madhur MS Lob HE McCann LA Iwakura Y Blinder Y Guzik TJ. Interleukin 17 promotes angiotensin II-induced hypertension and vascular dysfunction. Hypertension. (2010) 55:500–7. 10.1161/HYPERTENSIONAHA.109.145094 20038749 PMC2819301

[177] Norlander AE Saleh MA Kamat NV Ko B Gnecco J Zhu L. Interleukin-17A regulates renal sodium transporters and renal injury in angiotensin ii-induced hypertension. Hypertension. (2016) 68:167–74. 10.1161/HYPERTENSIONAHA.116.07493 27141060 PMC4900947

[178] Orejudo M García-Redondo AB Rodrigues-Diez RR Rodrigues-Díez R Santos-Sanchez L Tejera-Muñoz A. Interleukin-17A induces vascular remodeling of small arteries and blood pressure elevation. Clin Sci. (2020) 134:513–27. 10.1042/CS20190682 32104886

[179] Worthmann K Gueler F von Vietinghoff S Davalos-Mißlitz A Wiehler F Davidson A. Pathogenetic role of glomerular CXCL13 expression in lupus nephritis. Clin Exp Immunol. (2014) 178:20–7. 10.1111/cei.12380 24827905 PMC4360190

[180] He DN Chen WL Long KX Zhang X Dong GF. Association of serum CXCL13 with intrarenal ectopic lymphoid tissue formation in lupus nephritis. J Immunol Res. (2016) 2016:4832543. 10.1155/2016/4832543 27990444 PMC5136399

[181] Imaizumi T Aizawa T Segawa C Shimada M Tsuruga K Kawaguchi S. Toll-like receptor 3 signaling contributes to the expression of a neutrophil chemoattractant, CXCL1 in human mesangial cells. Clin Exp Nephrol. (2015) 19:761–70. 10.1007/s10157-014-1060-4 25471749

[182] De Palma G Castellano G Del Prete A Sozzani S Fiore N Loverre A. The possible role of ChemR23/Chemerin axis in the recruitment of dendritic cells in lupus nephritis. Kidney Int. (2011) 79:1228–35. 10.1038/ki.2011.32 21346723

[183] Moreth K Brodbeck R Babelova A Gretz N Spieker T Zeng-Brouwers J. The proteoglycan biglycan regulates expression of the B cell chemoattractant CXCL13 and aggravates murine lupus nephritis. J Clin Invest. (2010) 120:4251–72. 10.1172/JCI42213 21084753 PMC2993585

[184] Chung ACK Lan HY. Chemokines in renal injury. J Am Soc Nephrol. (2011) 22:802–9. 10.1681/ASN.2010050510 21474561

[185] Wen Z Xu L Xu W Yin Z Gao X Xiong S. Interleukin-17 expression positively correlates with disease severity of lupus nephritis by increasing anti-double-stranded DNA antibody production in a lupus model induced by activated lymphocyte derived DNA. PLoS ONE. (2013) 8:e58161. 10.1371/journal.pone.0058161 23472149 PMC3589375

[186] Dorraji SE Hovd A-MK Kanapathippillai P Bakland G Eilertsen GØ Figenschau SL. Mesenchymal stem cells and T cells in the formation of tertiary lymphoid structures in lupus nephritis. Sci Rep. (2018) 8:7861. 10.1038/s41598-018-26265-z 29777158 PMC5959845

[187] DorrajiSEKanapathippillaiPHovdA-MKStenersrødMRHorveiKDUrsvikA. Kidney tertiary lymphoid structures in lupus nephritis develop into large interconnected networks and resemble lymph nodes in gene signature. Am J Pathol. (2020) 190:2203–25. 10.1016/j.ajpath.2020.07.01532818496

[188] Kang S Fedoriw Y Brenneman EK Truong YK Kikly K Vilen BJ. BAFF induces tertiary lymphoid structures and positions T cells within the glomeruli during lupus nephritis. J Immunol. (2017) 198:2602–11. 10.4049/jimmunol.1600281 28235864 PMC5360485

[189] Luo R Cheng Y Chang D Liu T Liu L Pei G. Tertiary lymphoid organs are associated with the progression of kidney damage and regulated by interleukin-17A. Theranostics. (2021) 11:117–31. 10.7150/thno.48624 33391465 PMC7681089

[190] Summers SA Odobasic D Khouri MB Steinmetz OM Yang Y Holdsworth SR. Endogenous interleukin (IL)-17A promotes pristane-induced systemic autoimmunity and lupus nephritis induced by pristane. Clin Exp Immunol. (2014) 176:341–50. 10.1111/cei.12287 24528105 PMC4008978

[191] Witowski J Ksiazek K Warnecke C Kuzlan M Korybalska K Tayama H. Role of mesothelial cell-derived granulocyte colony-stimulating factor in interleukin-17-induced neutrophil accumulation in the peritoneum. Kidney Int. (2007) 71:514–25. 10.1038/sj.ki.5002082 17228364

[192] Liu B Tan W Barsoum A Gu X Chen K Huang W. IL-17 is a potent synergistic factor with GM-CSF in mice in stimulating myelopoiesis, dendritic cell expansion, proliferation, and functional enhancement. Exp Hematol. (2010) 38:877–84.e1. 10.1016/j.exphem.2010.06.004 20600582

[193] Chan AJ Alikhan MA Odobasic D Gan PY Khouri MB Steinmetz OM. Innate IL-17A-producing leukocytes promote acute kidney injury via inflammasome and Toll-like receptor activation. Am J Pathol. (2014) 184:1411–8. 10.1016/j.ajpath.2014.01.023 24631024

[194] Shahrara S Pickens SR Mandelin AM 2nd Karpus WJ Huang Q Kolls JK. IL-17-mediated monocyte migration occurs partially through CC chemokine ligand 2/monocyte chemoattractant protein-1 induction. J Immunol. (2010) 184:4479–87. 10.4049/jimmunol.0901942 20228199 PMC2858914

[195] Ding Y Li J Wu Q Yang P Luo B Xie S. IL-17RA is essential for optimal localization of follicular Th cells in the germinal center light zone to promote autoantibody-producing B cells. J Immunol. (2013) 191:1614–24. 10.4049/jimmunol.1300479 23858031 PMC3819396

[196] Majumder S Amatya N Revu S Jawale CV Wu D Rittenhouse N. IL-17 metabolically reprograms activated fibroblastic reticular cells for proliferation and survival. Nat Immunol. (2019) 20:534–45. 10.1038/s41590-019-0367-4 30962593 PMC6519710

[197] Ma K Du W Xiao F Han M Huang E Peng N. IL-17 sustains the plasma cell response via p38-mediated Bcl-xL RNA stability in lupus pathogenesis. Cell Mol Immunol. (2020) 18:1739–50. 10.1038/s41423-020-00540-4 32917979 PMC8245411

[198] Liu L-L Qin Y Cai J-F Wang H-Y Tao J-L Li H. Th17/Treg imbalance in adult patients with minimal change nephrotic syndrome. Clin Immunol. (2011) 139:314–20. 10.1016/j.clim.2011.02.018 21450528

[199] Zhang C Xiao C Wang P Xu W Zhang A Li Q. The alteration of Th1/Th2/Th17/Treg paradigm in patients with type 2 diabetes mellitus: relationship with diabetic nephropathy. Hum Immunol. (2014) 75:289–96. 10.1016/j.humimm.2014.02.007 24530745

[200] Lee JW Bae E Kwon S-H Yu M-Y Cha R-H Lee H. Transcriptional modulation of the T helper 17/interleukin 17 axis ameliorates renal ischemia-reperfusion injury. Nephrol Dial Transplant. (2019) 34:1481–98. 10.1093/ndt/gfy370 30544214

[201] Lin F-J Jiang G-R Shan J-P Zhu C Zou J Wu X-R. Imbalance of regulatory T cells to Th17 cells in IgA nephropathy. Scand J Clin Lab Invest. (2012) 72:221–9. 10.3109/00365513.2011.652158 22276947

[202] Ma J Li YJ Chen X Kwan T Chadban SJ Wu H. Interleukin 17A promotes diabetic kidney injury. Sci Rep. (2019) 9:2264. 10.1038/s41598-019-38811-4 30783187 PMC6381173

[203] Kuo H-L Huang C-C Lin T-Y Lin C-Y. IL-17 and CD40 ligand synergistically stimulate the chronicity of diabetic nephropathy. Nephrol Dial Transplant. (2018) 33:248–56. 10.1093/ndt/gfw397 28339909

[204] Mehrotra P Patel JB Ivancic CM Collett JA Basile DP. Th-17 cell activation in response to high salt following acute kidney injury is associated with progressive fibrosis and attenuated by AT-1R antagonism. Kidney Int. (2015) 88:776–84. 10.1038/ki.2015.200 26200947 PMC4589446

[205] Wang Z Shi W Liang X Wang W Liang J. Association of interleukin 17/angiotensin II with refractory hypertension risk in hemodialysis patients. Afr Health Sci. (2016) 16:766–71. 10.4314/ahs.v16i3.17 27917210 PMC5111972

[206] Dong X Bachman LA Miller MN Nath KA Griffin MD. Dendritic cells facilitate accumulation of IL-17 T cells in the kidney following acute renal obstruction. Kidney Int. (2008) 74:1294–309. 10.1038/ki.2008.394 18974760 PMC2948974

[207] Ge S Hertel B Koltsova EK Sörensen-Zender I Kielstein JT Ley K. Increased atherosclerotic lesion formation and vascular leukocyte accumulation in renal impairment are mediated by interleukin-17A. Circ Res. (2013) 113:965–74. 10.1161/CIRCRESAHA.113.301934 23908345 PMC3848055

[208] Kim Y-G Kim E-Y Ihm C-G Lee T-W Lee S-H Jeong K-H. Gene polymorphisms of interleukin-17 and interleukin-17 receptor are associated with end-stage kidney disease. Am J Nephrol. (2012) 36:472–477. 10.1159/000343571 23147652

[209] Allen ME Rus V Szeto GL. Leveraging heterogeneity in systemic lupus erythematosus for new therapies. Trends Mol Med. (2021) 27:152–71. 10.1016/j.molmed.2020.09.009 33046407 PMC8667782

[210] Lee E Han JH Bang CH Yoo SA Han KD Kim H-N. Risk of end-stage renal disease in psoriatic patients: real-world data from a nationwide population-based cohort study. Sci Rep. (2019) 9:16581. 10.1038/s41598-019-53017-4 31719568 PMC6851155

[211] Wan J Wang S Haynes K Denburg MR Shin DB Gelfand JM. Risk of moderate to advanced kidney disease in patients with psoriasis: population based cohort study. BMJ. (2013) 347:f5961. 10.1136/bmj.f5961 24129480 PMC3805477

[212] Chi C-C Wang J Chen Y-F Wang S-H Chen F-L Tung T-H. Risk of incident chronic kidney disease and end-stage renal disease in patients with psoriasis: a nationwide population-based cohort study. J Dermatol Sci. (2015) 78:232–238. 10.1016/j.jdermsci.2015.03.012 25862150

[213] Mori S Yoshitama T Hirakata N Ueki Y. Prevalence of and factors associated with renal dysfunction in rheumatoid arthritis patients: a cross-sectional study in community hospitals. Clin Rheumatol. (2017) 36:2673–82. 10.1007/s10067-017-3804-5 28884373 PMC5681610

[214] Couderc M Tatar Z Pereira B Tiple A Gilson M Fautrel B. Prevalence of renal impairment in patients with rheumatoid arthritis: results from a cross-sectional multicenter study. Arthritis Care Res. (2016) 68:638–44. 10.1002/acr.22713 26314697

[215] Levy AR Szabo SM Rao SR Cifaldi M Maksymowych WP. Estimating the occurrence of renal complications among persons with ankylosing spondylitis. Arthritis Care Res. (2014) 66:440–5. 10.1002/acr.22176 24106183

[216] Xiao M Lv Q Zhang Y Tu L Yang M Lin Z. Spondyloarthritis patients suffer increased risk of renal complications compared with general population: a retrospective observational study. Front Pharmacol. (2019) 10:1073. 10.3389/fphar.2019.01073 31620002 PMC6759995

[217] Ye W Zhuang J Yu Y Li H Leng X Qian J. Gender and chronic kidney disease in ankylosing spondylitis: a single-center retrospectively study. BMC Nephrol. (2019) 20:457. 10.1186/s12882-019-1658-6 31818273 PMC6902329

[218] Biswas PS. IL-17 in renal immunity and autoimmunity. J Immunol. (2018) 201:3153–9. 10.4049/jimmunol.1801042 30455371 PMC6524787

[219] Rodrigues-Díez R Aroeira LS Orejudo M Bajo M-A Heffernan JJ Rodrigues-Díez RR. IL-17A is a novel player in dialysis-induced peritoneal damage. Kidney Int. (2014) 86:303–15. 10.1038/ki.2014.33 24552849

[220] Lei L Zhao C Qin F He Z-Y Wang X Zhong X-N. Th17 cells and IL-17 promote the skin and lung inflammation and fibrosis process in a bleomycin-induced murine model of systemic sclerosis. Clin Exp Rheumatol. (2016) 34 (Suppl. 100):14–22. 26750756

[221] Paquissi FC. Immunity and fibrogenesis: the role of Th17/IL-17 axis in HBV and HCV-induced chronic hepatitis and progression to cirrhosis. Front Immunol. (2017) 8:1195. 10.3389/fimmu.2017.01195 29033929 PMC5626935

[222] Curciarello R Docena GH MacDonald TT. The role of cytokines in the fibrotic responses in crohn's disease. Front Med. (2017) 4:126. 10.3389/fmed.2017.00126 28824915 PMC5545939

[223] Zhang H-J Zhang Y-N Zhou H Guan L Li Y Sun M-J. IL-17A promotes initiation and development of intestinal fibrosis through EMT. Dig Dis Sci. (2018) 63:2898–2909. 10.1007/s10620-018-5234-x 30097894

[224] Tan H-L Regamey N Brown S Bush A Lloyd CM Davies JC. The Th17 pathway in cystic fibrosis lung disease. Am J Respir Crit Care Med. (2011) 184:252–8. 10.1164/rccm.201102-0236OC 21474644 PMC3381840

[225] Beringer A Miossec P. IL-17 and IL-17-producing cells and liver diseases, with focus on autoimmune liver diseases. Autoimmun Rev. (2018) 17:1176–85. 10.1016/j.autrev.2018.06.008 30321671

[226] Ferrantelli E Liappas G Vila Cuenca M Keuning ED Foster TL Vervloet MG. The dipeptide alanyl-glutamine ameliorates peritoneal fibrosis and attenuates IL-17 dependent pathways during peritoneal dialysis. Kidney Int. (2016) 89:625–35. 10.1016/j.kint.2015.12.005 26880457

[227] Dong Z Lu X Yang Y Zhang T Li Y Chai Y. IL-27 alleviates the bleomycin-induced pulmonary fibrosis by regulating the Th17 cell differentiation. BMC Pulm Med. (2015) 15:13. 10.1186/s12890-015-0012-4 25888222 PMC4340860

[228] Gu L Deng W-S Sun X-F Zhou H Xu Q. Rapamycin ameliorates CCl4-induced liver fibrosis in mice through reciprocal regulation of the Th17/Treg cell balance. Mol Med Rep. (2016) 14:1153–61. 10.3892/mmr.2016.5392 27315465 PMC4940054

[229] Hsu H-C Yang P Wang J Wu Q Myers R Chen J. Interleukin 17-producing T helper cells and interleukin 17 orchestrate autoreactive germinal center development in autoimmune BXD2 mice. Nat Immunol. (2008) 9:166–75. 10.1038/ni1552 18157131

[230] Ghali JR O'Sullivan KM Eggenhuizen PJ Holdsworth SR Kitching AR. Interleukin-17RA promotes humoral responses and glomerular injury in experimental rapidly progressive glomerulonephritis. Nephron. (2017) 135:207–23. 10.1159/000453059 27941331

[231] Mitsdoerffer M Lee Y Jäger A Kim H-J Korn T Kolls JK. Proinflammatory T helper type 17 cells are effective B-cell helpers. Proc Natl Acad Sci USA. (2010) 107:14292–7. 10.1073/pnas.1009234107 20660725 PMC2922571

[232] Catar RA Chen L Cuff SM Kift-Morgan A Eberl M Kettritz R. Control of neutrophil influx during peritonitis by transcriptional cross-regulation of chemokine CXCL1 by IL-17 and IFN-γ. J Pathol. (2020) 251:175–86. 10.1002/path.5438 32232854

[233] Griffin GK Newton G Tarrio ML Bu D-X Maganto-Garcia E Azcutia V. IL-17 and TNF-α sustain neutrophil recruitment during inflammation through synergistic effects on endothelial activation. J Immunol. (2012) 188:6287–99. 10.4049/jimmunol.1200385 22566565 PMC3370121

[234] Roussel L Houle F Chan C Yao Y Bérubé J Olivenstein R. IL-17 promotes p38 MAPK-dependent endothelial activation enhancing neutrophil recruitment to sites of inflammation. J Immunol. (2010) 184:4531–7. 10.4049/jimmunol.0903162 20228195

[235] Herjan T Yao P Qian W Li X Liu C Bulek K. HuR is required for IL-17-induced Act1-mediated CXCL1 and CXCL5 mRNA stabilization. J Immunol. (2013) 191:640–9. 10.4049/jimmunol.1203315 23772036 PMC3722902

[236] Sun D Novotny M Bulek K Liu C Li X Hamilton T. Treatment with IL-17 prolongs the half-life of chemokine CXCL1 mRNA via the adaptor TRAF5 and the splicing-regulatory factor SF2 (ASF). Nat Immunol. (2011) 12:853–60. 10.1038/ni.2081 21822258 PMC3597344

[237] Garcia-Romo GS Caielli S Vega B Connolly J Allantaz F Xu Z. Netting neutrophils are major inducers of type I IFN production in pediatric systemic lupus erythematosus. Sci Transl Med. (2011) 3:73ra20. 10.1126/scitranslmed.3001201 21389264 PMC3143837

[238] Biswas PS Aggarwal R Levesque MC Maers K Ramani K. Type I interferon and T helper 17 cells co-exist and co-regulate disease pathogenesis in lupus patients. Int J Rheum Dis. (2015) 18:646–53. 10.1111/1756-185X.12636 25960196

[239] Brkic Z Corneth OBJ van Helden-Meeuwsen CG Dolhain RJEM Maria NI Paulissen SMJ. T-helper 17 cell cytokines and interferon type I: partners in crime in systemic lupus erythematosus? Arthritis Res Ther. (2014) 16:R62. 10.1186/ar4499 24598455 PMC4060204

[240] López P Rodríguez-Carrio J Caminal-Montero L Mozo L Suárez A. A pathogenic IFNα, BLyS and IL-17 axis in Systemic Lupus Erythematosus patients. Sci Rep. (2016) 6:20651. 10.1038/srep20651 26847824 PMC4742957

[241] Robert M Miossec P. Effects of interleukin 17 on the cardiovascular system. Autoimmun Rev. (2017) 16:984–91. 10.1016/j.autrev.2017.07.009 28705781

[242] Rees F Doherty M Grainge M Lanyon P Davenport G Zhang W. Burden of comorbidity in systemic lupus erythematosus in the UK, 1999-2012. Arthritis Care Res. (2016) 68:819–27. 10.1002/acr.22751 26473719

[243] Benagiano M Borghi MO Romagnoli J Mahler M Bella CD Grassi A. Interleukin-17/Interleukin-21 and interferon-γ producing T cells specific for β2 glycoprotein I in atherosclerosis inflammation of systemic lupus erythematosus patients with antiphospholipid syndrome. Haematologica. (2019) 104:2519–27. 10.3324/haematol.2018.209536 30872365 PMC6959190

[244] Chen S Shimada K Zhang W Huang G Crother TR Arditi M. IL-17A is proatherogenic in high-fat diet-induced and *Chlamydia pneumoniae* infection-accelerated atherosclerosis in mice. J Immunol. (2010) 185:5619–27. 10.4049/jimmunol.1001879 20935201 PMC3046880

[245] Pacifici R. The role of IL-17 and TH17 cells in the bone catabolic activity of PTH. Front Immunol. (2016) 7:57. 10.3389/fimmu.2016.00057 26925062 PMC4756106

[246] Leonardi C Matheson R Zachariae C Cameron G Li L Edson-Heredia E. Anti-interleukin-17 monoclonal antibody ixekizumab in chronic plaque psoriasis. N Engl J Med. (2012) 366:1190–9. 10.1056/NEJMoa1109997 22455413

[247] Glatt S Baeten D Baker T Griffiths M Ionescu L Lawson ADG. Dual IL-17A and IL-17F neutralisation by bimekizumab in psoriatic arthritis: evidence from preclinical experiments and a randomised placebo-controlled clinical trial that IL-17F contributes to human chronic tissue inflammation. Ann Rheum Dis. (2018) 77:523–32. 10.1136/annrheumdis-2017-212127 29275332 PMC5890624

[248] Mease PJ Genovese MC Greenwald MW Ritchlin CT Beaulieu AD Deodhar A. Brodalumab, an anti-IL17RA monoclonal antibody, in psoriatic arthritis. N Engl J Med. (2014) 370:2295–306. 10.1056/NEJMoa1315231 24918373

[249] Satoh Y Nakano K Yoshinari H Nakayamada S Iwata S Kubo S. A case of refractory lupus nephritis complicated by psoriasis vulgaris that was controlled with secukinumab. Lupus. (2018) 27:1202–6. 10.1177/0961203318762598 29523055

[250] van Vollenhoven RF Hahn BH Tsokos GC Wagner CL Lipsky P Touma Z. Efficacy and safety of ustekinumab, an IL-12 and IL-23 inhibitor, in patients with active systemic lupus erythematosus: results of a multicentre, double-blind, phase 2, randomised, controlled study. Lancet. (2018) 392:1330–9. 10.1016/S0140-6736(18)32167-6 30249507

[251] Dai H He F Tsokos GC Kyttaris VC. IL-23 limits the production of IL-2 and promotes autoimmunity in lupus. J Immunol. (2017) 199:903–10. 10.4049/jimmunol.1700418 28646040 PMC5526729

[252] Edwards LJ Mizui M Kyttaris V. Signal transducer and activator of transcription (STAT) 3 inhibition delays the onset of lupus nephritis in MRL/lpr mice. Clin Immunol. (2015) 158:221–30. 10.1016/j.clim.2015.04.004 25869298 PMC4465043

[253] Maeda K Kosugi T Sato W Kojima H Sato Y Kamimura D. CD147/basigin limits lupus nephritis and Th17 cell differentiation in mice by inhibiting the interleukin-6/STAT-3 pathway. Arthritis Rheumatol. (2015) 67:2185–95. 10.1002/art.39155 25891969

[254] Ripoll È de Ramon L Draibe Bordignon J Merino A Bolaños N Goma M. JAK3-STAT pathway blocking benefits in experimental lupus nephritis. Arthritis Res Ther. (2016) 18:134. 10.1186/s13075-016-1034-x 27278657 PMC4898357

[255] Zhou X Chen H Wei F Zhao Q Su Q Lei Y. α-mangostin attenuates pristane-induced lupus nephritis by regulating Th17 differentiation. Int J Rheum Dis. (2020) 23:74–83. 10.1111/1756-185X.13743 31769201

[256] Zhou X Chen H Wei F Zhao Q Su Q Liang J. 3-acetyloxy-oleanolic acid attenuates pristane-induced lupus nephritis by regulating Th17 differentiation. J Immunol Res. (2019) 2019:2431617. 10.1155/2019/2431617 31240232 PMC6556267

[257] Yin Y Choi S-C Xu Z Zeumer L Kanda N Croker BP. Glucose oxidation is critical for CD4+ T cell activation in a mouse model of systemic lupus erythematosus. J Immunol. (2016) 196:80–90. 10.4049/jimmunol.1501537 26608911 PMC4684991

[258] Cornaby C Elshikha AS Teng X Choi S-C Scindia Y Davidson A. Efficacy of the combination of metformin and CTLA4Ig in the (NZB × NZW)F1 mouse model of lupus nephritis. Immunohorizons. (2020) 4:319–31. 10.4049/immunohorizons.2000033 32540987

[259] Li W Qu G Choi S-C Cornaby C Titov A Kanda N. Targeting T cell activation and lupus autoimmune phenotypes by inhibiting glucose transporters. Front Immunol. (2019) 10:833. 10.3389/fimmu.2019.00833 31057554 PMC6478810

[260] Sun F Wang HJ Liu Z Geng S Wang HT Wang X. Safety and efficacy of metformin in systemic lupus erythematosus: a multicentre, randomised, double-blind, placebo-controlled trial. Lancet Rheumatol. (2020) 2:e210–6. 10.1016/S2665-9913(20)30004-7 38268156

[261] Zhao C Chu Y Liang Z Zhang B Wang X Jing X. Low dose of IL-2 combined with rapamycin restores and maintains the long-term balance of Th17/Treg cells in refractory SLE patients. BMC Immunol. (2019) 20:32. 10.1186/s12865-019-0305-0 31484501 PMC6727508

[262] Chu Y Zhao C Zhang B Wang X Wang Y An J. Restoring T-helper 17 cell/regulatory T-cell balance and decreasing disease activity by rapamycin and all-trans retinoic acid in patients with systemic lupus erythematosus. Lupus. (2019) 28:1397–406. 10.1177/0961203319877239 31551029

[263] Lai Z-W Kelly R Winans T Marchena I Shadakshari A Yu J. Sirolimus in patients with clinically active systemic lupus erythematosus resistant to, or intolerant of, conventional medications: a single-arm, open-label, phase 1/2 trial. Lancet. (2018) 391:1186–96. 10.1016/S0140-6736(18)30485-9 29551338 PMC5891154

[264] You G Cao H Yan L He P Wang Y Liu B. MicroRNA-10a-3p mediates Th17/Treg cell balance and improves renal injury by inhibiting REG3A in lupus nephritis. Int Immunopharmacol. (2020) 88:106891. 10.1016/j.intimp.2020.106891 32853927

[265] Seo Y Mun CH Park S-H Jeon D Kim SJ Yoon T. Punicalagin ameliorates lupus nephritis via inhibition of PAR2. Int J Mol Sci. (2020) 21:4975. 10.3390/ijms21144975 32674502 PMC7404282

[266] Shui B Xia W Wen C Ding X. Jieduquyuziyin prescription suppresses IL-17 production and Th17 activity in MRL/lpr mice by inhibiting expression of Ca(2+)/calmodulin-dependent protein kinase-4. J Nat Med. (2015) 69:349–57. 10.1007/s11418-015-0900-1 25821132

[267] Yang J Yang X Yang J Li M. Hydroxychloroquine inhibits the differentiation of Th17 cells in systemic lupus erythematosus. J Rheumatol. (2018) 45:818–26. 10.3899/jrheum.170737 29545450

[268] Silva JC da Mariz HA Rocha LF da Jr Oliveira PSS de Dantas AT Duarte ALBP. Hydroxychloroquine decreases Th17-related cytokines in systemic lupus erythematosus and rheumatoid arthritis patients. Clinics. (2013) 68:766–71. 10.6061/clinics/2013(06)07 23778483 PMC3674253

[269] von Vietinghoff S Ouyang H Ley K. Mycophenolic acid suppresses granulopoiesis by inhibition of interleukin-17 production. Kidney Int. (2010) 78:79–88. 10.1038/ki.2010.84 20375992

[270] Slight-Webb S Guthridge JM Chakravarty EF Chen H Lu R Macwana S. Mycophenolate mofetil reduces STAT3 phosphorylation in systemic lupus erythematosus patients. JCI Insight. (2019) 4:e124575. 10.1172/jci.insight.124575 30674728 PMC6413783

[271] Furie R Rovin BH Houssiau F Malvar A Teng YKO Contreras G. Two-year, randomized, controlled trial of belimumab in lupus nephritis. N Engl J Med. (2020) 383:1117–28. 10.1056/NEJMoa2001180 32937045

[272] Prete M Leone P Frassanito MA Desantis V Marasco C Cicco S. Belimumab restores Treg/Th17 balance in patients with refractory systemic lupus erythematosus. Lupus. (2018) 27:1926–35. 10.1177/0961203318797425 30180771

